# Landscape-level movement patterns by lions in western Serengeti: comparing the influence of inter-specific competitors, habitat attributes and prey availability

**DOI:** 10.1186/s40462-016-0082-9

**Published:** 2016-07-01

**Authors:** Andrew M. Kittle, John K. Bukombe, Anthony R. E. Sinclair, Simon A. R. Mduma, John M. Fryxell

**Affiliations:** Department of Integrative Biology, University of Guelph, 50 Stone Road East, Guelph, Ontario N1G 2W1 Canada; Tazania Wildlife Research Institute, P.O. Box 661, Arusha, United Republic of Tanzania; Biodiversity Research Centre, University of British Columbia, Vancouver, BC V6T 1Z4 Canada; Present address: The Wilderness &Wildlife Conservation Trust, 130 Reid Avenue, Colombo 04, Sri Lanka

**Keywords:** *Crocuta crocuta*, *Panthera leo*, Prey distribution, Prey vulnerability, Resource utilization, Seasonality, Spatial ecology

## Abstract

**Background:**

Where apex predators move on the landscape influences ecosystem structure and function and is therefore key to effective landscape-level management and species-specific conservation. However the factors underlying predator distribution patterns within functional ecosystems are poorly understood. Predator movement should be sensitive to the spatial patterns of inter-specific competitors, spatial variation in prey density, and landscape attributes that increase individual prey vulnerability. We investigated the relative role of these fundamental factors on seasonal resource utilization by a globally endangered apex carnivore, the African lion (*Panthera leo*) in Tanzania’s Serengeti National Park. Lion space use was represented by novel landscape-level, modified utilization distributions (termed “localized density distributions”) created from telemetry relocations of individual lions from multiple neighbouring prides. Spatial patterns of inter-specific competitors were similarly determined from telemetry re-locations of spotted hyenas (*Crocuta crocuta*), this system’s primary competitor for lions; prey distribution was derived from 18 months of detailed census data; and remote sensing data was used to represent relevant habitat attributes.

**Results:**

Lion space use was consistently influenced by landscape attributes that increase individual prey vulnerability to predation. Wet season activity, when available prey were scarce, was concentrated near embankments, which provide ambush opportunities, and dry season activity, when available prey were abundant, near remaining water sources where prey occurrence is predictable. Lion space use patterns were positively associated with areas of high prey biomass, but only in the prey abundant dry season. Finally, at the broad scale of this analysis, lion and hyena space use was positively correlated in the comparatively prey-rich dry season and unrelated in the wet season, suggesting lion movement was unconstrained by the spatial patterns of their main inter-specific competitors.

**Conclusions:**

The availability of potential prey and vulnerability of that prey to predation both motivate lion movement decisions, with their relative importance apparently mediated by overall prey abundance. With practical and theoretical implications, these results suggest that while top carnivores are consistently cognizant of how landscape features influence individual prey vulnerability, they also adopt a flexible approach to range use by adjusting spatial behaviour according to fluctuations in local prey abundance.

**Electronic supplementary material:**

The online version of this article (doi:10.1186/s40462-016-0082-9) contains supplementary material, which is available to authorized users.

## Background

The distribution and abundance of top predators on the landscape can exert profound influence on the distribution and abundance of their prey [1–3]. This in turn can impact predator-prey population dynamics [4, 5] as well as ecosystem structure as mediated by trophic cascades [6, 7]. Understanding the factors that drive apex predator space use therefore provides valuable insights into community structure and dynamics and is accordingly of fundamental importance for the management and future conservation of both top predators and the broader systems of which they are a part.

A basic precept of behavioral ecology is that natural selection should favor organisms that use landscapes in a way that maximize fitness [8]. For predators a basic component of fitness is the rate of individual prey capture, which is assumed to be enhanced in areas of high prey density [9]. Hence, at the broadest (regional species range) scale, the distribution of large carnivores is obviously determined by the availability of suitable prey [10, 11]. Within functional ecosystems however, the mechanism governing predator distribution is more elusive, with space use either dictated by areas of the landscape where prey are particularly abundant or spatial locations where individual prey capture is more efficient [12].

In multi-predator systems, the location of inter-specific competitors also can influence decisions on space use by carnivores [13] often with subsequent impacts on population dynamics [14–16]. Exploitative and interference competition are particularly widespread among African carnivores and may be fundamental to shaping distribution patterns [17]. Lions and hyenas are the most important predators, functionally and numerically, in many African systems [18] and are potentially strong direct competitors given that their diet and ecological range extensively overlap [19–21]. As a result the two species typically exhibit negative interactions in the form of direct aggression [22] and kleptoparasitism [23] as they compete for the same suite of prey resources [24]. Adding further complexity to this important inter-specific relationship, the relative status of these top carnivores is unclear with dominance appearing to be a function of prey availability within the shared ecosystem [25].

Here we use landscape-level seasonal lion and hyena space use metrics as well as unusually comprehensive prey abundance and distribution data to investigate the drivers of space use by lions in a multi-prey, migratory system in the Western Corridor of Serengeti National Park, Tanzania. Specifically, we ask whether the use of space by lions is primarily influenced by 1) spatial niche partitioning with their primary inter-specific competitor in the system, the spotted hyena, 2) attributes of the landscape that increase individual prey vulnerability, or 3) the direct availability of prey. Furthermore, since spatial and temporal heterogeneity in resource availability partially underlies seasonal shifts in organism distribution patterns [26], we ask whether the factors influencing predator space use differ between wet and dry seasons. Due to the annual migration of wildebeest across the Greater Serengeti Ecosystem, prey availability varies considerably in the Western Corridor [27]. We demonstrate that landscape features that increase individual prey’s vulnerability to predation are consistently influential predictors of lion range utilization whereas measures of general prey availability influence lion movement patterns only when overall prey abundance in the study area is high. We further show that at the broad scale of this analysis, lions are not employing spatial niche partitioning with respect to their primary competitor in the system, the spotted hyena.

## Methods

### Study area

The 25 000 km^2^ Serengeti ecosystem includes three distinct regions – the Serengeti Plains, the Western Corridor and the North [28]. The system structure is dominated by its rainfall regimen with the amount of rainfall following a south-east (500 mm) to north-west (1100 mm) gradient [29]. The wet season runs from November through May and dry season June through October [27].

**Fig. 1:**
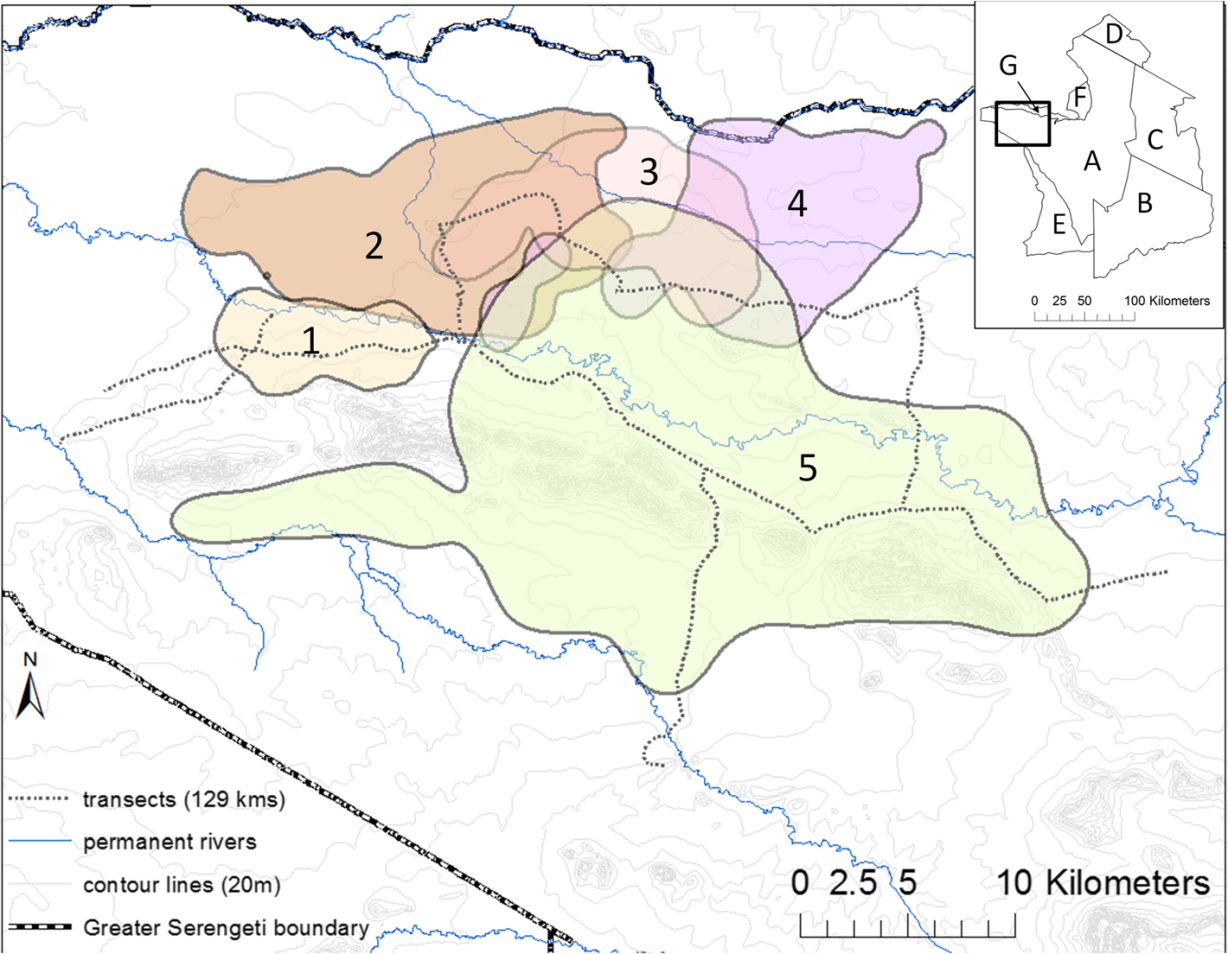
Study area in Serengeti National Park’s Western Corridor showing prey road strip transect locations and 95 % kernel density utilization distribution extents for five neighbouring lion prides (1 – 5) in 2010 wet season. Main rivers flowing East to West are Raho (*top*), Grumeti (*middle*) and Mbalageti (*bottom*). Inset shows Greater Serengeti Ecosystem with location of present study area indicated by black rectangle. A = Serengeti National Park, B = Ngorongoro Conservation Area, C = Loliondo Game Controlled Area, D = Masaai Mara National Reserve (Kenya), E = Maswa Game Reserve, F = Ikorongo Game Reserve and G = Grumeti Game Reserve

This study was conducted in a 1440 km^2^ portion of the Western Corridor (Fig. 1), a geologically complex region characterized by alluvial soil deposited by two major east-west oriented rivers, the Grumeti to the north and Mbalageti to the south, between which runs a series of Precambrian banded ironstone hills [27]. The Corridor is composed of a sparse woodland-grassland mosaic interspersed with patches of dense woodland [28]. This is a transitory zone for the wildebeest migration as it moves in a sweeping arc from the Serengeti Plains to the North, with the influx of migrating animals arriving in the Corridor at the onset of the dry season (June – July) and typically passing through prior to commencement of the wet season [30]. In contrast to the Serengeti Plains, the Western Corridor has substantial populations of resident ungulates [28], including resident wildebeest [31]. Lions and hyenas are the dominant predators in the Serengeti system occurring at densities of 0.12/km^2^ and 0.36/ km^2^ respectively [27] and accounting for ~85 % of large herbivore predation [20].

### Lion utilization distributions

Between December 2009 and June 2011 we attached GPS collars to a total of 6 adult female lions (Telonics TGW-4500, Mesa, AZ, USA) from 5 separate, adjacent prides in the central portion of the Western Corridor (Fig. 1). Lions were immobilized by veterinarians from Tanzania National Parks (TANAPA) and Tanzania Wildlife Research Institute (TAWIRI). Five GPS-telemetry collars were deployed at any one time and were programmed to record GPS locations every 2 h. Collar GPS fix success rate was 95.2 % ensuring no bias in telemetry re-locations [32]. Although un-collared prides occurred to the east, west and south of these 5 collared prides, there were no known un-collared prides within the focal study area. Pride 5 split shortly after collaring and this disruption is reflected by the subsequent wide-ranging movement of the collared lion from this pride (Fig. 1). This lion exhibited some overlap with an un-collared pride occupying the area immediately southeast of prides 1 and 2, as well as with other members of her original pride occupying areas southeast of pride 5’s core territory (Fig. 1).

We determined utilization distributions (cell size = 100x100m) for each individual for each season and year using fixed kernel density home range estimation (kde, adehabitatHR package in R) with reference bandwidth [33]. Although smoothing parameter or bandwidth selection critically impacts kernel analysis [33], particularly the determination of home range outer contours and to a lesser extent the estimation of the utilization distribution [34, 35], there is no single best method of choosing it a priori [36]. Unless data show a bivariate normal distribution, the reference bandwidth can over-smooth resulting in an UD larger than necessary [33]. However selecting the bandwidth that minimizes least-squares cross-validation [37], an alternative approach incorporated into most statistical software packages, tends to under-smooth when using large datasets (i.e. thousands of data points from telemetry re-locations) resulting in an unnecessarily restricted UD [33]. Alternative methods exist, such as “solve-the-equation plug in” [38] or reducing the reference bandwidth to a fixed [39] or even flexible [16] proportion, the use of which can be selected based on analysis requirements [33]. A central consideration in the current analysis was to maximize areas of overlap of inter-specific competitors, so the reference bandwidth was selected with the tradeoff of acknowledging that the larger UDs resulting might be less sensitive to detecting spatial patterns. We converted kernel UDs (kUDs) to volume UDs (vUDs) describing the percentage of the total territory that needs to be utilized for a given cell to be included [40]. We subtracted vUD values from 100 to arrive at a more intuitive value for each cell whereby low use cells get assigned low values and high use cells, high values (i.e. a cell with vUD of 75, which indicates that 75 % of the total range needs to be used to ensure that a lion uses this cell, becomes 100 - 75 = 25, which indicates that the probability of this cell being utilized by a lion is 0.25). To remove bias imposed by variation in territory size we integrated to 1 for each individual pride by dividing each cell value by the sum of all UD cell values for that pride.

We then transformed this probability of use value into a measure of localized density by multiplying cell values by lion group size for that season as determined during regular monitoring. All lion age and sex classes were included in pride size counts. If a pride changed composition during the course of a single season, verified by repeated observations of a new pride size, the size for that season was determined by averaging the distinct pride sizes weighted by the estimated seasonal duration of each pride size. These use layers represent lion spatial utilization but because they are weighted by group size are not true probability density functions and cannot therefore be termed utilization distributions in the formal sense [41]. Instead we consider these layers as localized density distributions (LDDs).

One pride – the Grumeti pride – contained two collared lions. These were spatially separated for prolonged periods (i.e. entire seasons) when one of the two females segregated herself from the pride to give birth, whereas the other female remained with the rest of the pride. We incorporated data from both individuals in order to represent more completely the full pride’s utilization of the landscape. To do this we calculated seasonal LDDs for each individual in the manner illustrated above and then joined the LDDs together by weighting each individual’s LDD values by the proportion of the total seasonal duration that it represented (i.e. if both individuals were tracked for the entire season, they were evenly weighted (50:50) but if one individual was tracked for 75 % of a given season and the other for the full season, weighting was 43:57). This method was followed for both dry and wet seasons.

Finally, we created seasonal landscape-level LDDs by amalgamating all individual pride LDDs for each season. Adjacent lion pride ranges often overlap [42], so if more than one pride territory overlapped a single cell on the landscape, we summed all values to determine the total seasonal use value for each cell each year. We had wet season data from two years (2010 and 2011) so in order to arrive at an overall wet season LDD we averaged the individual year LDDs by combining values across years and dividing by the number of years that a given cell was used. The final landscape-level LDDs used 7983 dry season relocations and 19164 wet season relocations from 6 individuals representing 5 prides.

The state (i.e. pregnant, hungry) as well as unique individual behavioural characteristics of animals within a population can result in individual variation in space use patterns [43] which may impact observed patterns at the population level [44, 45]. By collaring only adult females and weighting individual UDs by group size we attempted to account for some of this potential bias which is inherent in studies that use individual patterns to scale up to infer population-level processes. Sampling a single age/sex class has the effect of minimizing potential variation in behaviour driven by substantially different social roles and responsibilities [46]. Weighting individual UDs by group size works to buffer the effect, without discounting it, of collared individuals whose behavior, due to a specific state (e.g. pregnancy), circumstance (e.g. post-pride split) or individual specialization [44], varies markedly from that of their age/sex class and subsequently, social unit. Therefore weighting by group size should provide a more accurate reflection of the landscape utilization of the larger population.

### Inter-specific competition

During the same period as the lion tracking, we deployed GPS radio collars on 6 adult female hyenas from 5 separate clans that overlapped the home ranges of collared lion prides in the Western Corridor (LOTEK 3300 and 4400, Newmarket, ON, Canada and Tellus 2A, Followit, Lindemark, Sweden). Landscape-level space use layers were developed following the methods detailed above with relocations every 2 h, except hyena UDs were not weighted by clan size given that we were unable to effectively determine group sizes for all collared clans. Final landscape-level dry season UD was from 4328 relocations of 4 individuals from 4 clans and wet season UD was from 9669 relocations of 5 individuals from 5 clans.

### Prey availability

Across five days of every month for the duration of the study, we conducted a series of 6 transects throughout the study area between 6 am – noon. These encompassed periods of increased activity for most available potential prey species in the system [47–50]. Transects ranged in length from 9.4 to 43.6 km and comprised a total of 129 km of roads (Fig. 1). The distance of observed individuals from the transect line was determined using rangefinder binoculars. Beyond 100 m there is a decay in detection probability for ungulates in this system [51] so only those potential prey species (Table 1) [20, 52] within 100 m of the road were counted and their location along transects relative to the transect start point recorded using the vehicle odometer. Detection of individual animals was not affected by season [51] allowing for consistent comparisons between wet and dry seasons. The total area covered by these monthly transects was 129 km x 0.2 km = 25.8 km^2^. This exceptional dataset allowed tracking of both spatial and temporal prey distribution trends.

**Table 1:** Average adult female weights of lion prey species detected during monthly Western Corridor census surveys [98]

Common name	Scientific name	Weight (kg)
Giraffe	*Giraffa camelopardalis*	800
Buffalo	*Syncerus caffer*	450
Zebra	*Equus quagga*	250
Wildebeest	*Connochaetes taurinus*	170
Topi	*Damaliscus lunatus*	120
Waterbuck	*Kobus ellipsiprymnus*	180
Warthog	*Phacochoerus aethiopicusafricanus*	60
Grant’s gazelle	*Nanger granti*	55
Impala	*Aepyceros melampus*	50
Thomson’s gazelle	*Eudorcas thomsonii*	20
Olive baboon	*Papio anubis*	20

To determine overall prey availability, transects were overlaid with a non-overlapping sequence of 645 quadrats, each measuring 200 x 200 m. Quadrats were assigned two separate measures for each season: probability of occurrence of any prey species and average prey biomass. Probability of prey occurrence was a proportional measure of the number of monthly transects that a given transect quadrat was occupied by any potential prey divided by the total number of transects conducted for that season (i.e. if quadrat A was occupied by potential prey of any species during 3 dry season transects, and there were 5 dry season transects conducted, the probability of prey occurrence for this quadrat would be 3/5 = 0.6). This provides an indication of the reliability of a location in terms of the probability that it will contain prey for lions. Average biomass for each quadrat was calculated as the sum of all prey individuals in that quadrat multiplied by their species-specific weight (Table 1) and then divided by the number of transects conducted (i.e. if during the 5 dry season transects conducted, a given quadrat was detected to contain a combined total of 6 zebra and 10 wildebeest, the average biomass for that quadrat would be ((6 x 250 kg) + (10 x 170 kg))/5 = 640 kg). This measure provides information about the gross seasonal distribution and abundance of prey on the landscape. The correlation between prey abundance (sum of all prey individuals in each quadrat/number of transects conducted) and prey biomass was |r| > .9 for both seasons.

Quadrats were also characterized by their composition of four broad land cover categories based on Reed et al.’s physiognomic classifications [53], as well as proximity to water sources and proximity to ranger posts and/or tourist lodges. Land cover classes were open grassland, wooded grassland, open woodland and dense woodland. Open grassland was composed of grassed areas (2–100 %) with < 20 % shrub cover and < 2 % tree cover whereas wooded grasslands had similar shrub cover but tree cover between 2 and 19 %. Open woodland was comprised of 20–49 % shrubs or trees and dense woodland > 50 % shrub or tree coverage. Correlation analysis was conducted to ensure that variable collinearity does not bias statistical inference (|r| < 0.7) [54].

We used logistic regression appropriate for proportion data (generalized linear models with binomial error structure and logit link function in R) to determine the model that best explained the probability that quadrats were occupied by any prey [55, 56]. Hosmer and Lemeshow goodness-of-fit and Likelihood ratio tests were used to determine adequacy of model fit. To determine the average biomass/cell we conducted log-linear modeling using a negative binomial distribution and log link. A negative binomial distribution was chosen over Poisson due to over-dispersion of the data [56]. Model assumptions were verified by plotting residuals vs. fitted values and creating normal QQ plots.

Modeling of prey metrics was conducted by stepwise deletion of predictor variables, starting from the fully saturated (or global) model which included six variables – distance to water, distance to ranger posts and/or tourist lodges, proportion of open grassland, proportion of wooded grassland, proportion of open woodland, proportion of dense woodland plus a quadratic term (Distance_water^2^). The quadratic was included based on the expectation that many prey species have non-linear associations with water (e.g. need to be close to water to drink but not too close due to increased risk of predation). Stepwise deletion was conducted using Likelihood ratio tests, which are appropriate to compare between nested models [57]. Predictors were retained in the final model when their P-values were <0.2 to prevent the inadvertent omission of important variables [58]. The backward stepwise variable elimination process, though widely utilized in ecological modeling [57, 59] can be considered inferior to the protocol of determining alternative plausible models and then challenging the data with these models to see which the data best supports [60]. When considering competing models representing separate underlying hypotheses stepwise approaches are flawed, however the current goal was to employ a set of variables carefully selected a priori based on biological considerations and determine the best available combination of them to explain prey occurrence and abundance patterns across the landscape. As such, we feel this approach was justified.

To verify adequacy of the resultant best models (Table 2), we tested for spatial autocorrelation in model residuals, first by creating a bubble plot (“sp” package in R) which plots model residuals vs. spatial coordinates, to qualitatively evaluate whether similarly valued residuals were clumped [57] (Additional file 1: Figure S1). We then used variograms (“gstat” package in R; Additional file 2: Figure S2) to quantitatively verify that spatial autocorrelation was not an issue in the models [57]. As variograms assume isotrophy, we also plotted multi-directional variograms to verify this assumption [57] (Additional file 3: Figure S3). The best models were then used to map the probability of seasonal prey occurrence and average prey biomass across the landscape. Output cell size was 200 X 200 m to match the size of input prey transect quadrats. We further evaluated model fit by plotting observed values for each of our prey availability measures against the values projected from final models and determining the resultant correlation coefficients. These ranged from |r| = 0.11 for average prey biomass in the dry season to 0.28 for frequency of occurrence in the wet season indicating a weak effect size and suggesting that some other, unquantified variable(s) were influencing prey distribution in the study area (Additional file 4: Figure S4 and Additional file 5: Figure S5). Finally, we conducted sensitivity analysis of these best models by plotting the projected seasonal average biomass and frequency of occurrence against each individual input variable comprising each top model and estimating correlation coefficients. This provides a visual means to indicate the relative influence of individual explanatory variables (Additional file 6: Figure S6, Additional file 7: Figure S7 and Additional file 8: Figure S8).

**Table 2:** Best seasonal models explaining the frequency of occurrence and average biomass of prey

Season	Response	Predictor variables	θ	SE	*P*-value
Dry	Frequency of occurrence	Distance to permanent water	-2.49E-04	1.11E-04	<0.05
		(Distance to permanent water)^2^	4.67E-08	2.18E-08	<0.05
		Distance to rangerpost/lodge	1.66E-05	9.30E-06	<0.1
		Wooded grassland	2.31E-01	1.18E-01	<0.1
Dry	Average biomass	Distance to rangerpost/lodge	-7.91E-05	1.79E-05	<0.0001
Wet	Frequency of occurrence	Distance to water	5.04E-04	2.02E-04	<0.05
		(Distance to water)^2^	-3.42E-07	1.10E-07	<0.01
		Distance to rangerpost/lodge	-5.44E-05	7.88E-06	<0.0001
		Wooded grassland	-1.60E-01	9.81E-02	<0.2
		Dense woodland	-4.61E-01	2.05E-01	<0.05
Wet	Average biomass	Distance to rangerpost/lodge	-5.18E-05	1.77E-05	<0.01
		Open grassland	1.37E + 00	2.16E-01	<0.0001
		Open woodland	1.60E + 00	4.23E-01	<0.001

Based on 200 x 200 m prey transect quadrats (*n* = 645). All models determined from backward stepwise elimination procedure using likelihood ratio tests, starting from full model (*k* = 7)

### Landscape attributes

The distance to drainage beds with clearly defined embankments and mean percentage of woody cover greater than 0.4 m high were used to characterize potential lion hunting cover [59, 61]. Embankments were defined by Classes 1 – 3 of the RiversV3 shapefile in the Serengeti Database www.serengetidata.org whereas cover was based on the average amount of woody cover calculated from each of the 27 physiognomic land-cover classes identified by Reed et al. [53] with the height based on minimum cover requirements for lions [62, 63]. We also characterized the landscape in terms of the straight line distance to nearest water sources as measured using GIS analysis tools. This included all rivers and ephemeral streams in the wet season (Class 1 - 4) but since most water sources in the Western Corridor are highly seasonal, only distance to permanent water (Class 1 and 2) was measured in the dry season. Two permanent waterholes recently dug by Tanzania National Park (TANAPA) staff were added to GIS layers separately. The distance to permanent water sources and distance to embankments were highly correlated (|r| > 0.7) so in the dry season only the distance to water variable was maintained. Landscape topography can impact resource selection for a variety of large mammals [64–67] so we created a digital elevation model (DEM) raster from the Serengeti contour layer from which we determined the average elevation and slope across the study area.

### Modeling lion space use

We created a rectangular grid of 5760 cells, each measuring 500 x 500 m, across the study area, as defined by the outer margins of the largest 95 % landscape-level seasonal LDD. Each grid cell was then populated with the average lion use value from the landscape-level seasonal LDDs as well as the eight independent variables representing our three hypotheses (Table 3).

**Table 3:** Independent variables representing each of the three hypotheses proposed to explain lion space use

Hypothesis	Abbreviation	Variables
Inter-specific competition	INTER	log(Hyena UD value)
Landscape attributes	LAND	distance to permanent water (dry) or all water (wet)
		distance to embankment (wet season only)
		cover %
		elevation
		log(slope)
Prey availability	PREY	average prey biomass
		probability of prey use

The proportion of the seasonal lion LDDs included in each analysis was constrained by the need to overlap with the landscape-level hyena UDs from the same season. This reduced the number of 500 x 500 m grid cells included in the analysis from 1986 to 629 in the dry season and from 3722 to 1405 in the wet season. We felt this reduction was warranted in that we wanted to compare lion use directly to contemporary hyena utilization as this parallel tracking of the region’s two primary predators was one of the key attributes of the research.

To account for the spatial autocorrelation in the response variable, we used generalized least squares (GLS) mixed effect regression models with an explicit correlation structure (the random effects) to determine the influences of lion space use in our study area [57]. We log-transformed the response variable (average lion LDD value), the inter-specific competition variable (average hyena UD value) and average slope in order to comply with model assumptions. To determine the appropriate correlation structure for the data we ran a saturated model (including all predictor variables) with different correlation structures (our random effects) using the restricted maximum likelihood method (REML) [57]. We used AIC to select the most appropriate correlation structure (rational quadratic, corRatio in R) and variograms to verify that spatial autocorrelation was adequately accounted for [56].

We then conducted two separate modeling procedures, the first to determine the best model for each of our three hypotheses (INTER = inter-specific competition, LAND = landscape attributes, PREY = prey availability) and the second to compare those best models, and their additive combinations, to investigate the relative influence of each on lion landscape utilization. To determine the best model for each hypothesis we created model sets of all hypothesis-specific potential predictor variables (Table 3) and used an information theoretic approach using ΔAIC to evaluate and rank models. Single parameter models with ΔAIC values <2 were considered superior to multi-parameter top ranked models [68]. Model fit was verified by plotting normalized residuals against fitted values and investigating residual distribution. Once we had determined the best model for each hypothesis for each season, we created a suite of eight competing models including the null, the three best single hypothesis models and all additive combinations. We compared hypotheses using AIC and Akaike weights (*w*_*i*_) to determine the weight of evidence in support of each [68]. To directly compare the relative influence of our three hypotheses we summed *w*_*i*_ of all models in which each hypothesis was represented, ensuring equal representation for valid comparisons [68]. To investigate the influence and association of individual parameters we re-ran all models using REML to ensure unbiased parameter estimates for each [57] and used model averaging, a form of multi-model inference, to determine final unbiased estimates with unconditional confidence intervals [68]. Model fit was further investigated by determining the correlation coefficient of the log of observed lion space use and the log of use projected from final models, as well as from visual comparisons of observed utilization maps and those projected from model output.

Statistical and spatial analysis was undertaken using R software version 2.15.1 [69], ArcMap 10.1 [70] and Geospatial Modeling Environment 0.7.2.1 [71].

### Direct lion observations

From January 2010 through June 2011 collared lions were regularly re-located on the ground and observed from a jeep for a total of 649.5 h. This included 232 observations < 30 min in duration and 198 monitoring periods of individual lions where observation duration was ≥ 30 min. Between June 2010 and June 2011 we conducted 177 individual follows of radio-collared lions amounting to 607.5 h of monitoring. The average duration of observations was 3.4 h (range 0.5 – 19.5) with 332.5 h occurring during the day (7:00 – 18:00), 210.9 h at night (19:00 – 6:00) and 64.1 h during crepuscular periods (6:00 – 7:00 and 18:00 – 19:00). Most nocturnal observations occurred during 10 extended day-night follows of collared individuals during the 48 h surrounding the full moon. These extended follows were conducted monthly between June 2010 and May 2011 with the exception of December 2010 and January 2011. Lions were observed with the naked eye when moonlight was sufficient and otherwise with night-vision binoculars, occasionally supplemented with a hand-held, red-filtered spotlight. The seasonal breakdown saw 306.5 h of monitoring in the dry season and 301 h in the wet season.

## Results

**Fig. 2:**
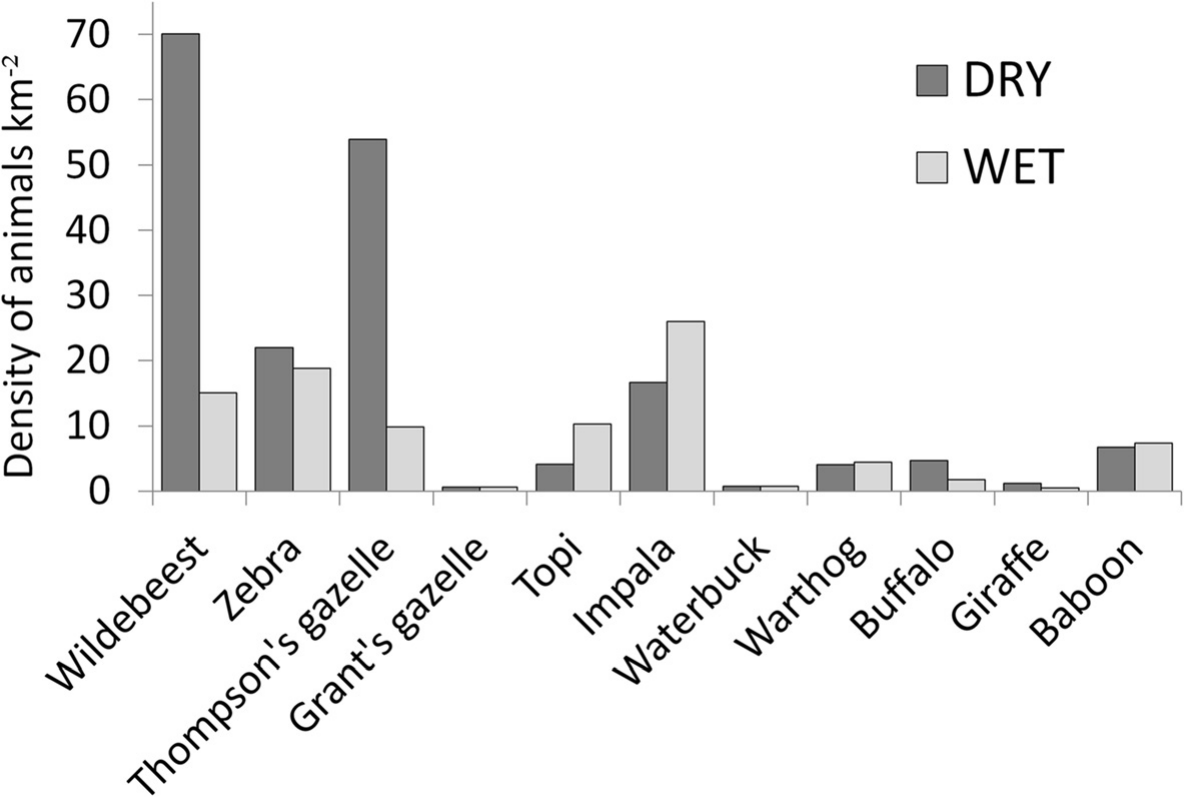
Seasonal density of selected prey species (#/km^2^) as determined from total animals observed during monthly (*N* = 18) road strip transects (129 km x 200 m). Assumes all animals within 100 m of transect were detected

**Fig. 3:**
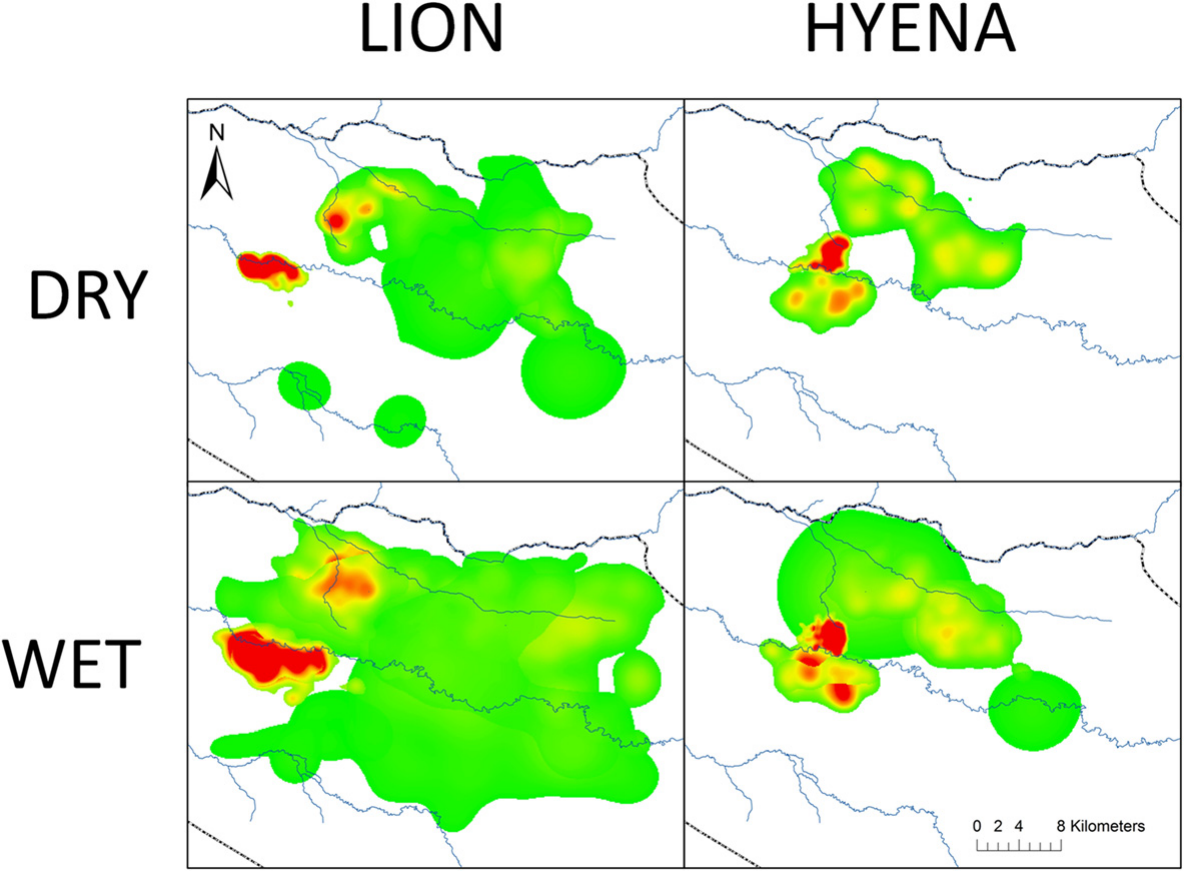
Seasonal landscape-level 95 % utilization distributions (UDs) for lions and hyenas. Serengeti National Park boundary is shown as a thick *black and white line* and permanent rivers as *blue lines*. UDs transition from low use (*green*) to high use (*red*). Lion dry season UD (*top left*) represents 7983 relocations from 5 lions in 4 prides; lion wet season UD (*bottom left*) represents 19164 relocations from 6 lions in 5 prides; hyena dry season UD (*top right*) represents 4328 relocations from 4 hyenas in 4 clans; and hyena wet season UD (*bottom right*) represents 9669 relocations of 5 hyenas from 5 clans

Density estimates based on monthly transect data clearly show the increased dry season availability of potential lion prey species, particularly migrant wildebeest and Thomson’s gazelles (Fig. 2). Landscape level lion density distribution maps reflect the increased importance of permanent water sources in this season, whereby lion range utilization can be seen to contract in their vicinity (Fig. 3, left panels). This pattern is not observed for hyena utilization distributions (Fig. 3, right panels).

Model evaluation resulted in single variable models representing each hypothesis in both seasons (Table 4; See Additional file 9: Table S1 for full model competition results). This ensures that hypothesis comparisons are not beholden to representative models with substantially different numbers of parameters, allowing greater confidence in the validity of comparisons.

**Table 4:** Model-averaged coefficient estimates with unbiased standard errors for final seasonal model variables for each hypothesis. Model averaging utilized all models (*N* = 8) included in the final model suite

Season	Hypothesis	Variables	θ	SE
DRY	INTER	log(hyenaUD)^a^	0.107592	0.046941
	LAND	distance_permanent_h2o^a^	-0.000193	0.000074
	PREY	Average biomass^a^	0.003274	0.001067
WET	INTER	log(hyenaUD)	-0.014632	0.025151
	LAND	distance_embankment^a^	-0.000102	0.000048
	PREY	Frequency	0.460227	0.526398

^a^ = 95 % Confidence Intervals do not overlap 0

**Fig. 4:**
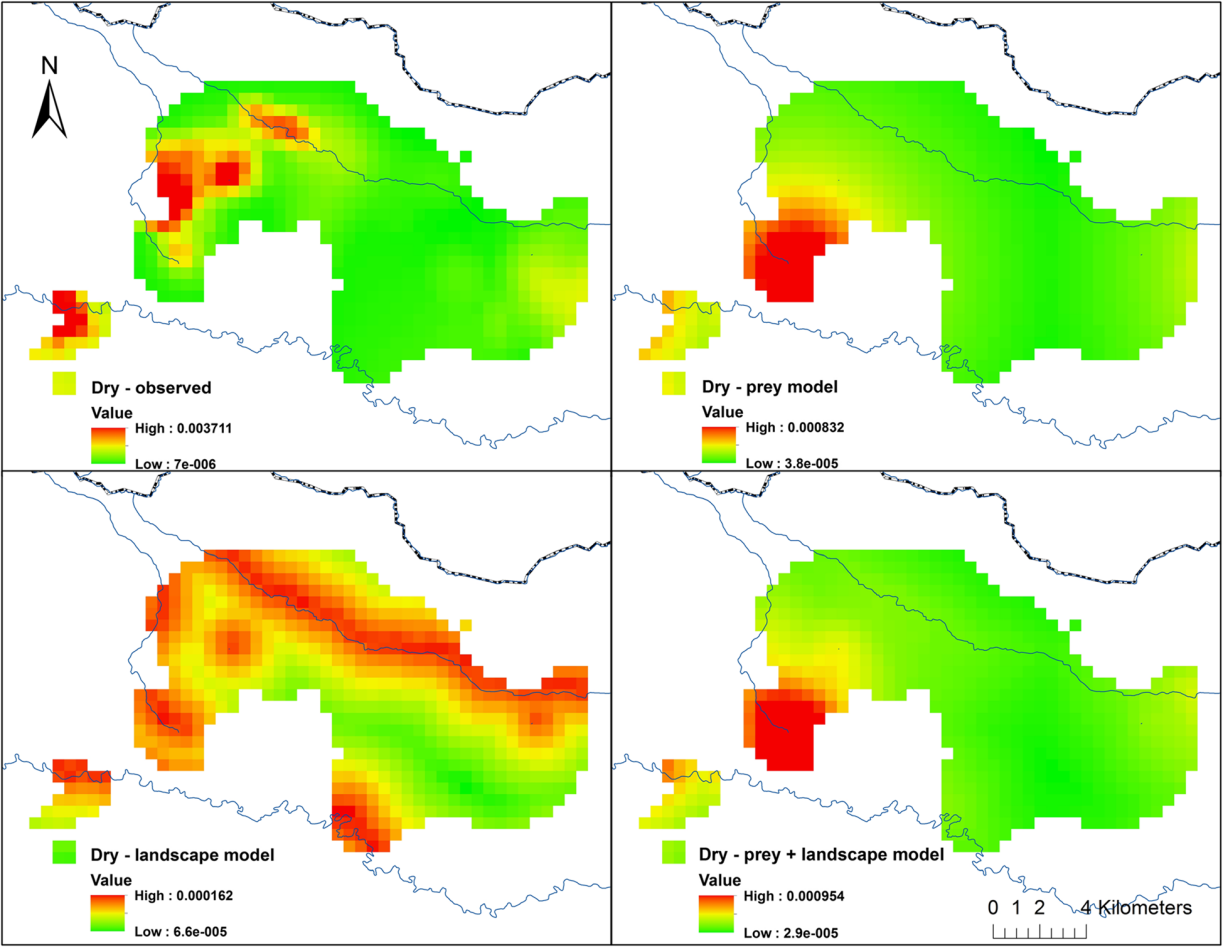
Observed and model-projected dry season lion utilization distributions in Serengeti National Park’s western corridor. *Top left* shows observed lion use; *top right* shows lion use projected from the prey availability model; *bottom left* shows lion use projected from the landscape attributes model; and *bottom right* shows lion use projected from the prey availability + landscape attribute model. *Dark line* at top is the National Park boundary and *blue lines* are permanent rivers. Displayed lion UDs are only those portions of total lion ranges that overlap hyena UDs

**Fig. 5:**
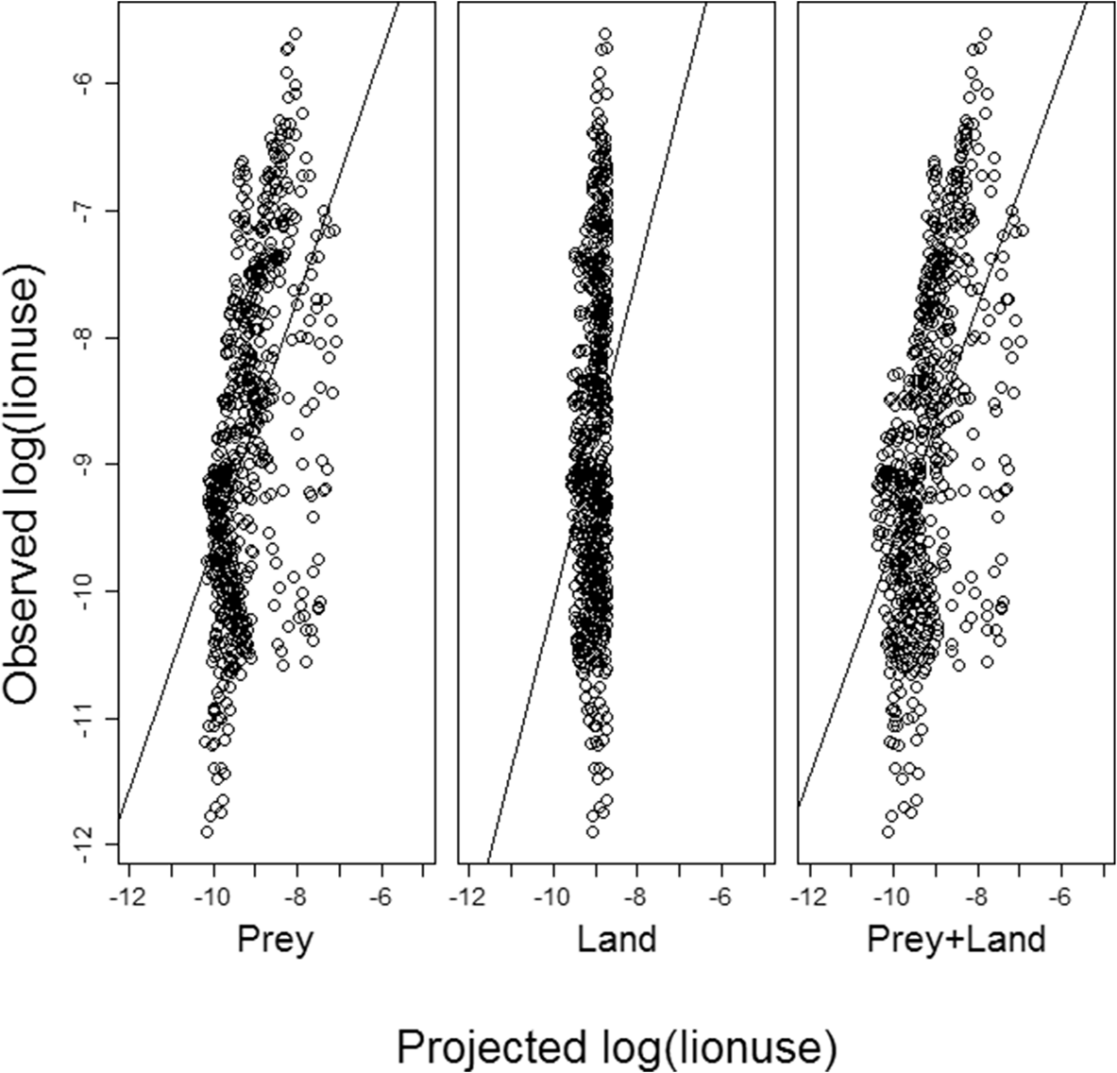
Correlation between observed and projected dry season lion use. Correlation between log of observed lion dry season space use (i.e. the probability of occupancy of a quadrat) and (*left*) log of prey availability model-projected dry season lion space use (|r| = 0.33); (*middle*) log of landscape attribute model-projected dry season lion space use (|r| = 0.26); and (*right*) log of prey availability + landscape attribute model-projected dry season lion space use (|r| = 0.35)

**Fig. 6:**
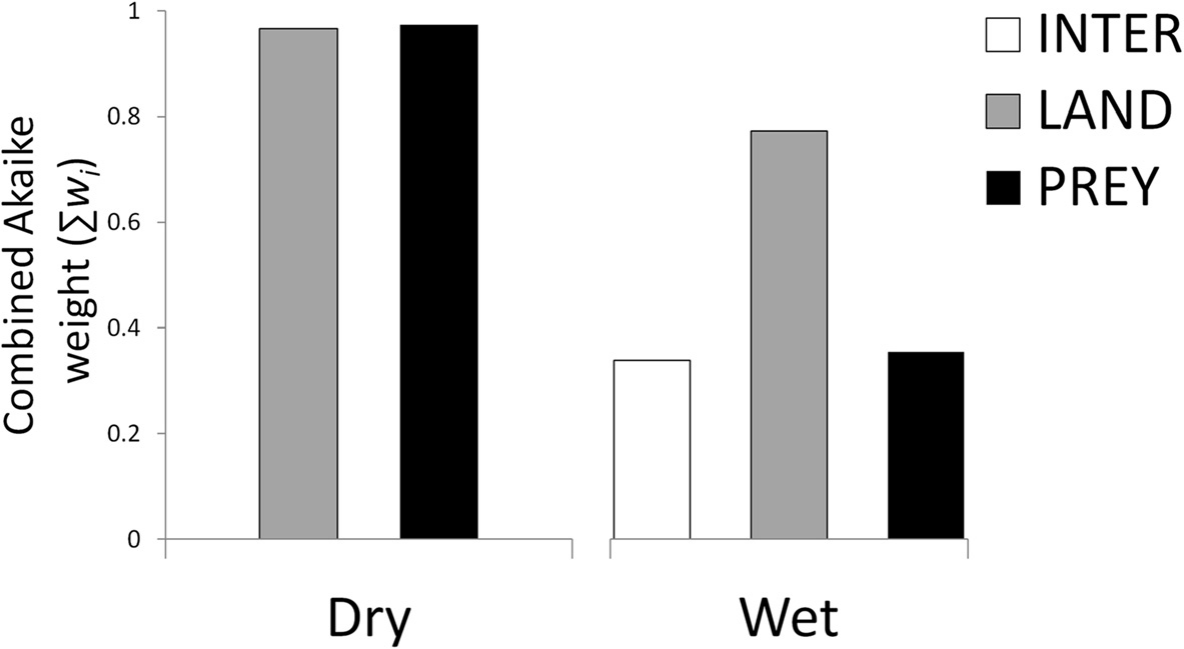
The relative influence of each hypothesis on lion space use by season. Summed Akaike weights (*w*_*i*_) across all models representing each hypothesis (INTER = inter-specific competition, LAND = landscape attributes, PREY = prey availability; *n* = 2 in dry season and 4 in wet season) and indicating the relative influence of each hypothesis on lion space use by season. INTER hypothesis is not considered in the dry season since association between lion and hyena use was positive for this season rendering the inter-specific competition hypothesis untenable

In the dry season, localized lion density was concentrated in areas of the landscape that were also heavily used by hyenas (Table 4). The model that best explained landscape-level lion space use during this season included elements of all three hypotheses. However, a positive association with hyenas indicates that lion spatial utilization patterns were not being influenced by spatial separation from their primary inter-specific competitor in this season, which was the expectation if inter-specific competition, acting through spatial niche partitioning, was driving lion space use. Therefore this hypothesis was eliminated from further consideration in this season and analysis reduced to a direct competition between prey resources and landscape attributes. The resulting best dry season model included both prey availability and landscape attributes (Table 5). Assessment of model fit also indicated that the combined model showed the highest correlation between observed and projected lion use (Figs. 4 and 5). Cumulative Akaike weighting suggested that prey availability and landscape attributes were almost equally associated with how lions utilize space in this season (Fig. 6) although prey biomass exerted the greater influence in the top model (Additional file 10: Figure S9). Specifically, in addition to areas of high hyena use, lion space use was concentrated during the dry season close to permanent water where prey biomass is high (Table 4).

**Table 5:** Model comparison table showing ΔAIC, Akaike weights (*w*_*i*_) and ranking for dry and wet seasons

	Dry season			Wet season		
Model	ΔAIC	*w*_*i*_	Rank	ΔAIC	*w*_*i*_	Rank
NULL	13.16	0.0013	4	2.24	0.1059	4
INTER	NA	NA	NA	3.65	0.0522	6
LAND	7.21	0.0249	3	0.00	0.3240	1
PREY	5.41	0.0611	2	3.70	0.0511	7
INTER + LAND	NA	NA	NA	1.34	0.1660	3
INTER + PREY	NA	NA	NA	5.14	0.0248	8
LAND + PREY	0.00	0.9128	1	1.14	0.1836	2
INTER + LAND + PREY	NA	NA	NA	2.51	0.0925	5

INTER hypothesis is not considered in the dry season since association between lion and hyena use was positive for this season rendering the inter-specific competition hypothesis untenable

**Fig. 7:**
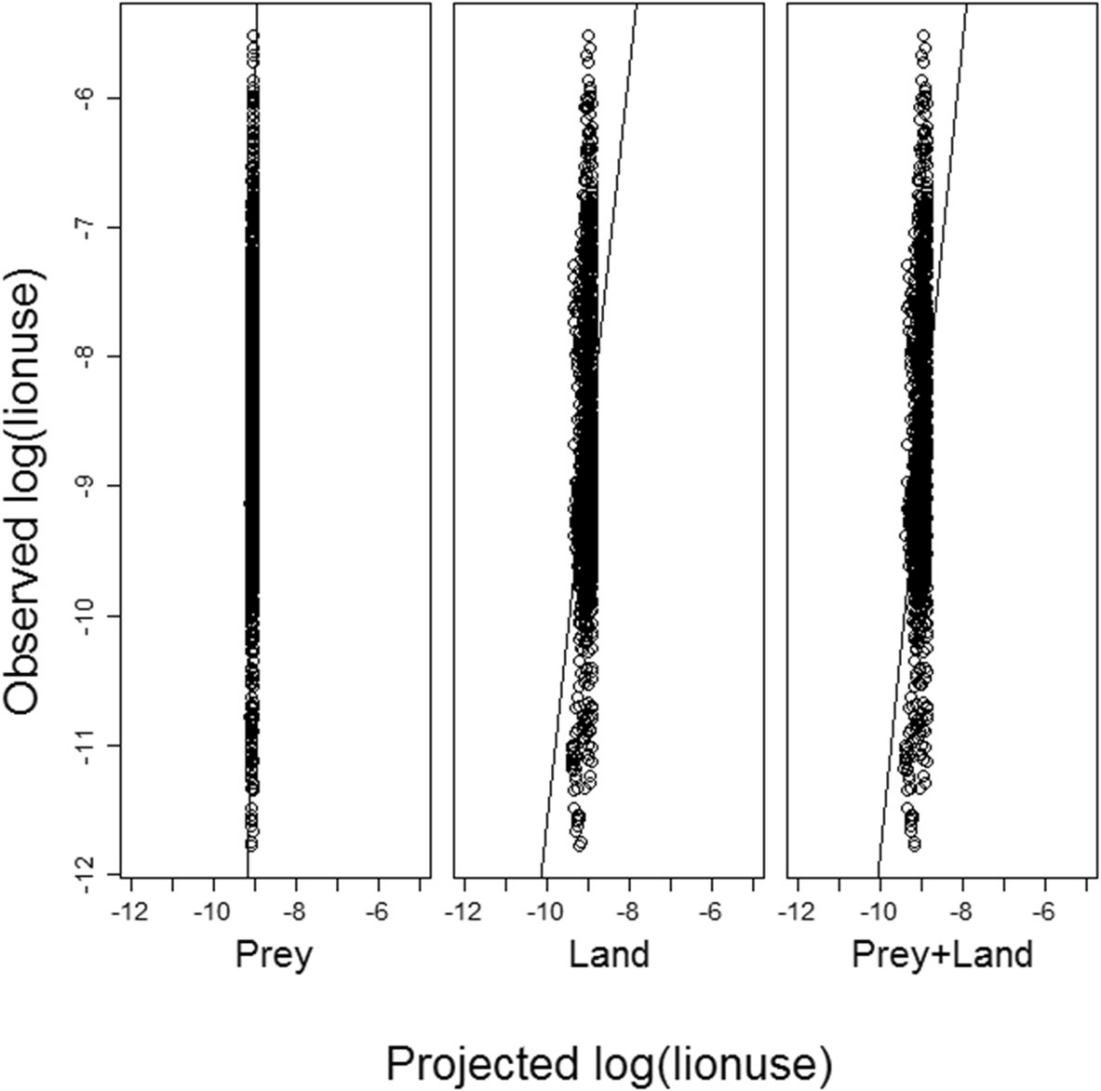
Correlation between observed and projected wet season lion use. Correlation between log of observed lion wet season space use (i.e. the probability of occupancy of a quadrat) and (*left*) log of prey availability model-projected wet season lion space use (|r| = 0.37); (*middle*) log of landscape attribute model-projected wet season lion space use (|r| = 0.18); and (*right*) log of prey availability + landscape attribute model-projected wet season lion space use (|r| = 0.23)

**Fig. 8:**
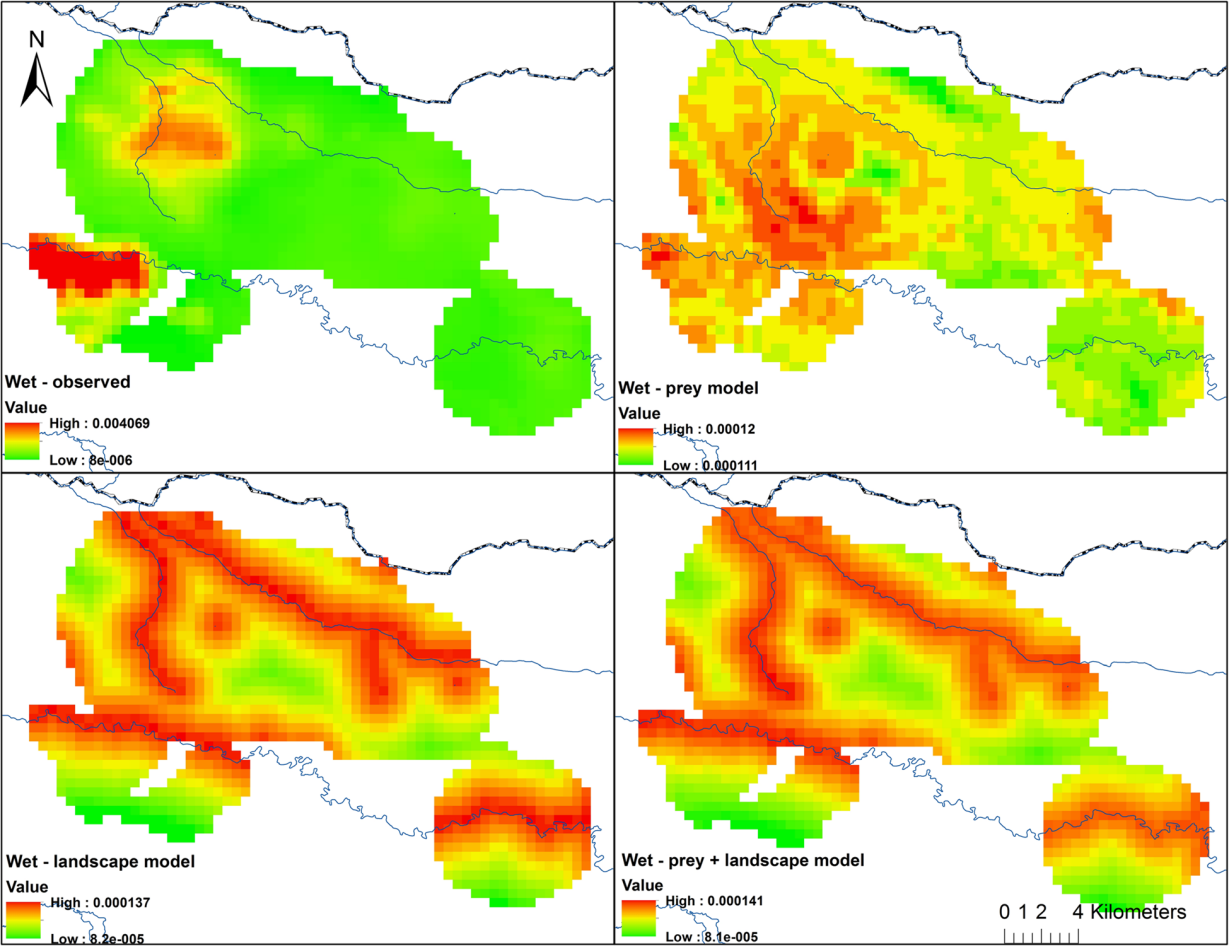
Observed and model-projected wet season lion utilization distributions (500 m grid) in Serengeti National Park’s western corridor. *Top left* shows observed lion use; *top right* shows lion use projected from the prey availability model; *bottom left* shows lion use projected from the landscape attributes model; and *bottom right* shows lion use projected from the prey availability + landscape attribute model. *Dark line* at *top* is the National Park boundary and *blue lines* are permanent rivers. Displayed lion UDs are only those portions of total lion ranges that overlap hyena UDs

The pattern was quite different in the wet season with cumulative model weighting suggesting landscape attributes influenced lion movement patterns more than twice as much as either inter-specific competition or prey availability (Fig. 6). During this season, localized lion density was disproportionately concentrated in areas in close proximity to embankments but their distribution was not associated with prey availability or hyena spatial utilization (Tables 4 and 5). Despite this, there was a higher correlation between observed lion use and use projected from the best prey availability model than from either the landscape attribute or combined model (Fig. 7). The narrow range of projected lion use values however suggests that additional, un-quantified factors are influencing lion movement during this season of prey scarcity. This is reflected in the maps of observed vs. projected use (Fig. 8).

## Discussion

In Serengeti’s Western Corridor the massive influx of migrant herbivores arrives during the dry season so prey abundance for lions is considerably more plentiful than during the wet season (Fig. 2). This increased seasonal abundance is reflected in the movement patterns of individual lions, which undertake fewer long range (>500 m) movements between 2-hourly telemetry relocations during the dry season than the wet season, both in the day and at night (Additional file 11: Figure S10). Given the sheer mass of prey that enters the Western Corridor at this time, it is perhaps not surprising that apex predators are cuing in on them and that during this season 71 % of all lion kills (*n* = 55) were wildebeest. Predictability is aided by the physiological limitations of the migrants, since grazers such as wildebeest, zebra and Thomson’s gazelle need to regularly drink and are therefore constrained in their dry season distribution by the availability of water [72, 73]. During the dry season available water sources are limited as the vast majority of streams and small water holes disappear, and even the two major rivers – the Grumeti and Mbalageti - dry up into a series of unconnected, stagnating pools. These two factors – that migrating herbivores need to regularly drink and that there are few places on the landscape where this is possible – work in favour of the region’s top predators, allowing them to adopt area-restricted search behaviour to take advantage of aggregated prey [74]. Lions in the semi-arid savanna in Zimbabwe similarly focus their movement in proximity to waterholes where they move at slower speeds and use more tortuous paths [75]. Thus it seems that water is the “spatial anchor” (sensu [12]) that allows predators to “win” the behavioural response race during the dry season.

The two lion prides that we were able to observe most frequently and for prolonged periods spent many daytime dry season hours in close proximity to the few remaining water sources in their territories, presumably waiting for the arrival of the migrant herds of wildebeest and zebra (Additional file 12: Figure S11). At these times, pride members displayed little regard for concealment, instead positioning themselves such that lions were often placed directly between prey herds and the water. On several occasions this strategy resulted in multiple kills for each pride. Perhaps lions were simply taking advantage of the physiological limitations of migrants that are most thirsty and therefore driven to drink during the heat of the day. However, herbivores can make behavioural adjustments to reduce predation risk at important, spatially fixed resources like waterholes [76], so despite the apparent success of the predators here, perhaps the tendency for migrants to arrive at water sources en masse in the middle of the day can reduce per capita prey risk due to dilution effects [77] and/or predator confusion [78] during the time when lions are typically least active [20] (Additional file 13: Figure S12).

The larger watercourses in the Western Corridor represent not only a source of drinking water for migrants, but also obstacles that must be crossed in order to continue toward the main dry season grazing areas to the north [79]. Just as there were few accessible drinking pools available to herbivores in the dry season there are also limited river sections that can be easily crossed due to thick vegetation and steep banks. Flat, shallow river segments therefore become high density thoroughfares for migrant herbivores for a few crucial dry season weeks, encouraging predators to remain in close proximity.

Lions did not avoid those parts of the landscape utilized by their main inter-specific competitor, the spotted hyena, and were in fact strongly positively associated with areas of high hyena utilization in the prey-rich dry season. There are three plausible explanations for the observed association between competitors: a) lions were cuing in on areas of high hyena use, b) areas of high lion use were being tracked by hyenas, or c) lions and hyenas were independently selecting the same locations of high prey availability. Hyenas are coursing predators so are unlikely to select for the same landscape features as lions for hunting purposes which hints at the probability that the observed positive association resulted from one species tracking the other. However, over the course of 650 h of direct lion observation spread throughout the year, including long periods of sustained individual follows, we observed only nine interactions with hyenas. Five of these were aggressive encounters, of which four were over kills. During these aggressive events, hyenas supplanted 2 – 3 female lions with cubs twice and single male lions supplanted groups of hyenas twice. The relative paucity of aggressive interactions suggests that these competitors were not actively tracking one another, suggesting that food is not a limiting resource for either species during the dry season. Both lions [13, 80] and hyenas [81] select for areas of high prey abundance at intermediate scales (i.e. 3^rd^ order [82]). Perhaps inter-specific competition is of limited concern during the dry season because migratory prey is plentiful, so top predators independently utilized similar, prey-rich areas. However, competition avoidance can be a more subtle process than a lack of obvious interactions. Both Hopcraft et al. [61] and Davidson et al. [80] in their analyses of lion predation events observed scale-dependent kill site selection with broader scale lion distribution influenced by prey abundance and finer scale prey utilization (i.e. 4^th^ order [82]) predominantly influenced by habitat features that increase prey vulnerability. Given that lions and hyenas employ divergent hunting techniques, a finer scale of analysis could potentially detect more subtle spatial separation between these predators within these wider shared regions that is not apparent here [83]. Neither hyenas nor lions are clearly sub-ordinate to the other, with interaction outcomes typically dependent on relative numbers and group composition [23]. Therefore unlike subordinate cheetahs (*Acinonyx jubatus*) which actively avoid both lions [13, 84] and hyenas [85], or leopards (*P. pardus*), which avoid dominant lions [13] and tigers (*P. tigris*) [86], avoidance behaviour is not entrenched here and in the absence of intense competition, was not observed.

In the wet season, overall prey biomass is much lower in the Western Corridor and given the widespread availability of both forage and water, less predictable spatially. At this time, with relatively few prey and few limiting resources, lions may be unable to effectively track where prey are most abundant. Alternately, given that prey herds congregate in the open grasslands during this season [51] (Table 2) it may be more difficult for lions to access individual prey there due to close grouping [87–89] and open habitat [90] promoting improved predator detection. As a result, lions are cuing in on areas of the landscape that should increase individual prey vulnerability, disproportionately utilizing areas in proximity to embankments which allow effective concealment and thus offer the potential to increase hunting success [61]. During this season zebra comprised 48 % of lions kills (*n* = 21) with buffalo (19 %), wildebeest (19 %) and warthog (14 %) also important.

**Fig. 9:**
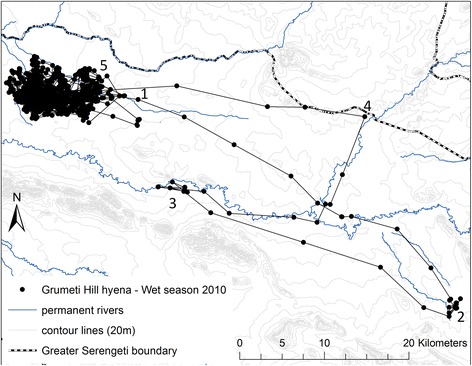
Extra-territorial movements of Grumeti Hill hyena during the late wet season 2010. Large cluster of *black circles* in *upper left* represents Wet season range with 19 day “commute” visible to the Musabi Plains in the southeast. Point 1 = departure from usual range (May 07, 18:00), 2 = first extra-territorial cluster 55 km from centre of usual range (May 8, 22:00 – May 12, 18:00), 3 = second extra-territorial cluster along Grumeti river (May 13, 04:00 – May 24, 20:00), 4 = travel along boundary of protected area complex (May 25, 18:00), and 5 = return to usual range (May 26, 02:00). Minimum travel distance based on summed step lengths = 160 km

We saw no correlation between lion and hyena space use during the wet season, despite the decrease in available prey. As coursing predators, hyenas are unlikely to bias their space use towards ambush features such as embankments which might de-couple their movements from those of lions. Additionally, hyenas in the Serengeti are unusual (but see [91]), in that they can undertake extended extra-territorial commutes to access areas of increased prey density [92]. One of the collared hyenas in this study undertook such a commute in the late wet season when prey availability in the Corridor was low, moving > 50 km southeast over the course of 19 days, presumably to access migrants on their way west (Fig. 9). Perhaps this ability to move beyond territorial boundaries relieves some of the burden of food acquisition which might otherwise increase competitive interactions with inter-specifics, promoting co-existence.

Overall, the observed variation in lion range use was not well captured by the best models, in the wet season in particular, as evidenced from the narrow range of predicted lion utilization values (Figs. 5 and 7). This appears to suggest that the model parameterization was suboptimal or that other factors that were not the focus of this study influence lion movement decisions.

One potential shortfall in model parameterization might stem from the employment of daytime prey transects. Savanna ungulates have been observed to alter their habitat preferences according to time of day [93] so the reliance here on daytime transects might limit our ability to detect the full range of lion prey distribution. Lions in the study area did hunt diurnally as well as nocturnally, with the hourly observed probability of a hunt, based on 52 observed hunting episodes, 0.078 in the day (7:00 – 18:00), 0.109 during crepuscular periods (6:00 – 7:00 and 18:00 – 19:00) and 0.057 at night (19:00 – 6:00). While this partially validates the reliance on daytime transects, most nocturnal observations in this study were undertaken during full moon periods when hunting success, if not effort, is lower [94]. Additionally, lions displayed a higher frequency of long range movement (>500 m) during the night than during the day across both wet and dry seasons (Additional file 11: Figure S10 and Additional file 13: Figure S12), which is indicative of increased nocturnal activity and presumably increased hunting effort. Furthermore, lions in the dry season were more frequently farther from water sources nocturnally than during the day (Additional file 12: Figure S11). At night ungulates are less likely to visit waterholes due to increased predation risk [76] forcing lions to similarly move away from these resources to access prey. These nocturnal behavioural changes have the potential to further alter prey distribution patterns [1]. Therefore, it is recommended that future work incorporate methods to determine nocturnal prey distributions in order to arrive at a more complete understanding of the processes discussed here.

A key element that might have weakened the observed relationships between lion space use and the variables that were considered here is the behavioural state of individual lions in the study. Of the 6 radio-collared female lions, 5 of them had cubs during the course of the research. In lion society females typically retreat from the main pride to give birth and can stay separated from their pride for several weeks after cubs are born [20]. During this period these lions can alter space use decisions based on the prioritized need to keep cubs secure from con- and inter-specifics. In our study area females post-partum ranged widely in behavior. One female moved to the periphery of her pride’s range and remained separate from her pride for several months whereas another, in the same pride, withdrew to a secluded location within the central portion of her pride’s range and resumed movement with the pride only a few weeks after the birth of her cubs. Yet another female gave birth shortly after her initial pride split and she remained alone with her cubs, wandering widely for almost a year before settling into a new pride near the end of the study period. In such a scenario it is likely that modelling each collared lion’s resource utilization would have resulted in the detection of a wide range of influential factors depending on the individual and a general synthesis of broad-scale space use patterns would not have been possible. Using the admittedly more complex amalgamation method that we employed here allows individual differences to be incorporated into model structure but still manages to synthesize these differences and detect, albeit weakly, selection processes that are driving the broad-scale patterns observed.

A tradeoff here is that we use model outputs, which already represent approximations of the processes that are the focus of those models, both as response and independent variables. The “noise” created by this strategy necessarily dilutes our ability to detect subtle effects but is compensated for by the ability to perceive the overarching drivers of landscape-level use emerging from such a complex system, defined by multiple prides of varying size and structure. Understanding these broad patterns was the foremost goal of the research and that model results concurred with expectations based on extensive direct observations of lions within this system serves to further validate the use of this procedure.

One possible extension of this novel landscape-level analysis is the creation of a layer of predator space use that can spread beyond the boundaries of the original source area (assuming new locations allow the accurate measurement of necessary co-variates) to predict predator distribution. This modelling process has been successfully used in this way to link habitat quality to carnivore range size [95] and to create broad-scale predation risk layers to which potential prey species respond [96, 97].

## Conclusions

Our results clearly suggest that both overall prey availability and landscape features that increase individual prey vulnerability influence space use decisions by Serengeti lions in the Western Corridor. Avoidance of main inter-specific competitors was not observed, suggesting that broad scale lion space use decisions are fundamentally shaped by the need to locate, secure and capture prey. The relative contribution of these prey-based factors varies seasonally and appears to hinge on the overall abundance of prey within the region as well as its predictability, ensuring that when prey are scarce habitat features promoting hunting success become relatively more influential. This underscores the flexible approach to range use employed by top carnivores and highlights the importance of investigating a multi-faceted suite of ecological variables when the goal is to understand the drivers of carnivore landscape utilization. In multi-predator assemblages where prey availability varies seasonally, as is the case in most tropical and sub-tropical systems, these results have important management implications. Finally, top predators are essential in shaping the trophic structure of the ecosystem but are some of the most imperiled of all species on earth [7]. These results show that conservation of predators, and the whole trophic cascade, requires a knowledge of the fundamental factors that motivate their utilization of the landscape.

## Additional files

Additional file 1: Figure S1. Bubble plots of top model standardized residuals vs. spatial coordinates for A. average prey biomass (kg/km^2^) in the dry season, B. average biomass (kg/km^2^) in the wet season, C. frequency of prey occurrence in the dry season, and D. frequency of prey occurrence in the wet season. Residual values are distinguished by colour with negative values in red and positive values in green. Excessive clumping of similar values (i.e. red clumps vs green clumps) indicates possible spatial autocorrelation, which appears absent from these plots. (GIF 70 kb) Additional file 2: Figure S2. Variograms showing variance of top model standardized residuals for A. average prey biomass (kg/km^2^) in the dry season, B. average biomass (kg/km^2^) in the wet season, C. frequency of prey occurrence in the dry season, and D. frequency of prey occurrence in the wet season. (GIF 40 kb) Additional file 3: Figure S3. Multi-directional variograms of standardized residuals for A. average prey biomass (kg/km^2^) in the dry season, B. average biomass (kg/km^2^) in the wet season, C. frequency of prey occurrence in the dry season, and D. frequency of prey occurrence in the wet season. Directions are indicated by degree values 0 = North-South, 45 = Northeast-Southwest, 90 = East-West and 135 = Southeast-Northwest. From the lack of strong spatial patters it appears that isotrophy is a reasonable assumption. (GIF 64 kb) Additional file 4: Figure S4. Correlation between log of observed prey biomass (kg/km^2^) for each quadrat in the prey transects (*N* = 645) and (left) log of model-projected dry season prey biomass from the best dry season model (|r| = 0.11) and (right) log of model-projected wet season prey biomass (|r| = 0.15) from the best wet season model. (TIFF 1235 kb) Additional file 5: Figure S5. Correlation between observed frequency (i.e. average probability that a quadrat is occupied by prey) for each quadrat in the prey transects (*N* = 645) and (left) log of model-projected dry season prey frequency from the best dry season model (|r| = 0.12) and (right) log of model-projected wet season prey frequency (|r| = 0.28) from the best wet season model. (TIFF 1235 kb) Additional file 6: Figure S6. Sensitivity analysis of input variables comprising the best wet season prey biomass model. The left graph shows prey biomass (kg/km^2^) values projected from the top model (Distance to disturbance + Open grassland + Open woodland) against the distance to disturbance input variable values (|r| = -0.46). The middle graph shows the same projected biomass values against the proportion of open grassland input variable values (|r| = 0.76). The right graph shows the same model output on the Y-axis against the proportion of open woodland input variable values (|r| = 0.06). (TIFF 1235 kb) Additional file 7: Figure S7. Sensitivity analysis of input variables comprising the best dry season prey frequency model. The left graph shows prey frequency (average probability of occurrence) values projected from the top model (Distance to permanent water + Distance to permanent water^2^ + Distance to disturbance + Wooded grassland) against the distance to permanent water input variable values (|r| = 0.06). The middle graph shows the same projected frequency values against the distance to disturbance input variable values (|r| = 0.38). The right graph shows the same model output on the Y-axis against the proportion of wooded grassland input variable values (|r| = 0.59). (TIFF 1235 kb) Additional file 8: Figure S8. Sensitivity analysis of input variables comprising the best wet season prey frequency model. The far left graph shows prey frequency (average probability of occurrence) values projected from the top model (Distance to all water + Distance to all water^2^ + Distance to disturbance + Wooded grassland + Dense woodland) against the distance to permanent water input variable values (|r| = -0.20). The inside left graph shows the same projected frequency values against the distance to disturbance input variable values (|r| = -0.87). The inside right graph shows the same model output on the Y-axis against the proportion of wooded grassland input variable values (|r| = -0.08). The far right graph shows the same projected frequency values against the proportion of dense woodland input variable values (|r| = -0.35). (TIFF 1235 kb) Additional file 9: Table S1. Results of competition to determine best model for each hypothesis (INTER, LAND and PREY) for each Season (DRY and WET). Best models as determined by ΔAIC are highlighted using bold text. Where ΔAIC < 2 the model with the fewest parameters was selected as the best model. (XLSX 12 kb) Additional file 10: Figure S9. Sensitivity analysis of input variables comprising the best dry season lion space use model. The left graph shows log(lionuse) values projected from the top model (Average prey biomass + Distance to permanent water) against the average prey biomass input variable values. The right graph shows the same model output on the Y-axis against the distance to permanent water input variable values. Both the visual pattern and the Pearson correlation coefficients (|r| = 0.89 and |r| = -0.33 respectively) indicate that average prey biomass is the more influential variable. (TIFF 1235 kb) Additional file 11: Figure S10. Histograms showing 2 h step lengths (m) for all radio-collared lions (*N* = 6) in the study area between December 2009 and June 2011. The y-axis is a measure of relative frequency of occurrence so that seasons with different numbers of step lengths can be compared. In both the day (6:00 – 18:00) and night (18:00 – 6:00) lions moved longer distances more frequently in the wet season than the dry season with diurnal mean movement distance = 105 m during the dry season and 135 m during the wet season (*t* = -5.1; *P* < 0.0001) and nocturnal mean movement distance = 449 m during the dry season and 604 m during wet season (*t* = -10.5; *P* < 0.0001). During both diurnal and nocturnal periods 2-h movement distances were usually below 500 m. (TIFF 367 kb) Additional file 12: Figure S11. Histograms showing average distance to water (m) for Kirawira pride between December 2009 and June 2011. The y-axis is a measure of the relative frequency of occurrence so that seasons with different numbers or re-locations can be compared. In both the wet and dry seasons lions preferred to be in close proximity to water sources. This preference was stronger in the daytime than nocturnally in both seasons, with the average dry season daytime distance = 463 m and average nighttime distance = 549 m, *t* = -3.314, *P* < 0.001) and average wet season daytime distance = 341 m and average nighttime distance = 386 m, *t* = -4.632, *P* < 0.0001). Pride lions were within 100 m of water more than 2x as frequently in the dry season daytime that dry season night. (PNG 4 kb) Additional file 13: Figure S12. Histograms showing 2 h step lengths (m) for all radio-collared lions (*N* = 6) in the study area between December 2009 and June 2011. The y-axis is a measure of relative frequency of occurrence so that seasons with different numbers of step lengths can be compared. In both the wet and dry seasons lions moved longer distances more frequently at night than during the day with wet season mean movement distance = 135 m during the day and 604 m during the night (*t* = -52.1; *P* < 0.0001) and dry season mean movement distance = 105 m during the day and 449 m during the night (*t* = -29; *P* < 0.0001). In both seasons 2-h movement distances were usually below 500 m. (TIFF 367 kb)
