# Supplementary figures and images for: Landscape-level movement patterns by lions in western Serengeti: comparing the influence of inter-specific competitors, habitat attributes and prey availability
Source: Mov Ecol. 2016 Jul 1;4:17. doi: 10.1186/s40462-016-0082-9 (PMC4929767; doi:10.1186/s40462-016-0082-9)

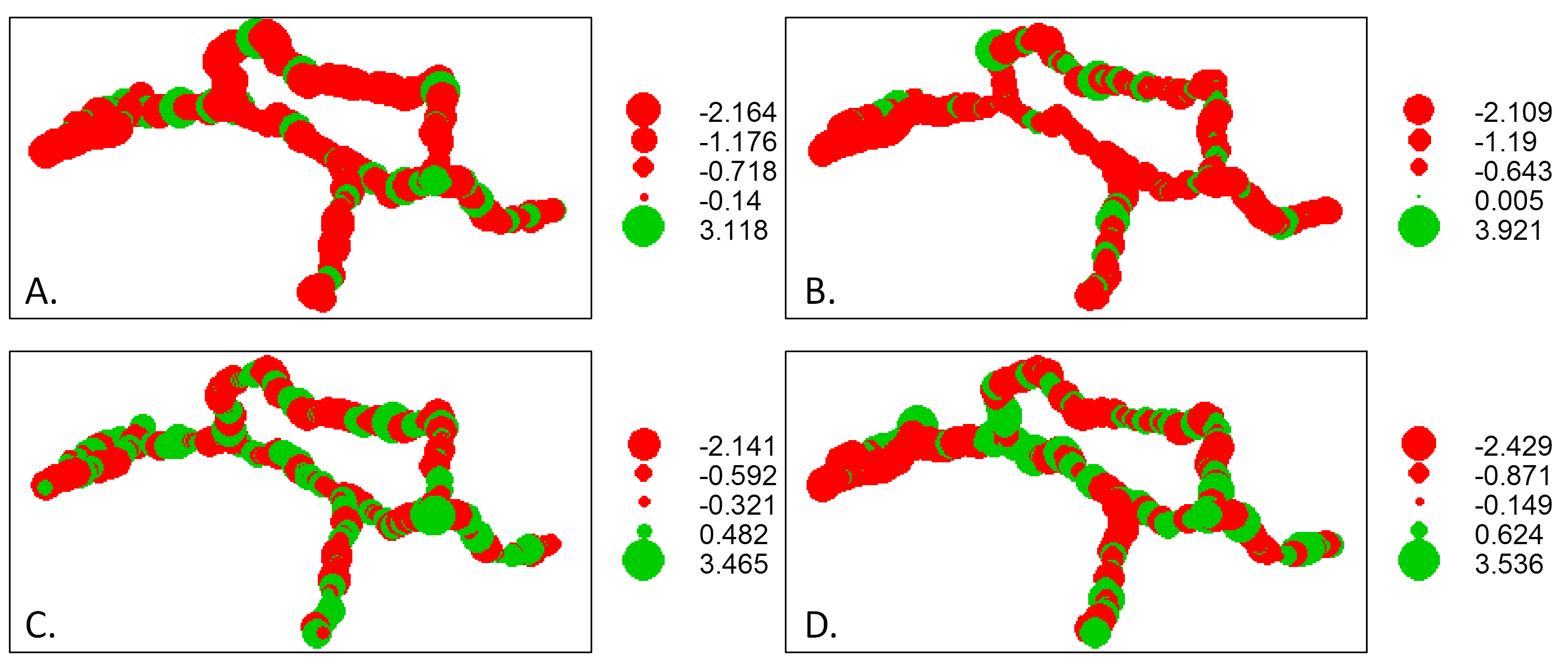

Supplement: Additional file 1: Figure S1. — Bubble plots of top model standardized residuals vs. spatial coordinates for A. average prey biomass (kg/km2) in the dry season, B. average biomass (kg/km2) in the wet season, C. frequency of prey occurrence in the dry season, and D. frequency of prey occurrence in the wet season. Residual values are distinguished by colour with negative values in red and positive values in green. Excessive clumping of similar values (i.e. red clumps vs green clumps) indicates possible spatial autocorrelation, which appears absent from these plots. (GIF 70 kb) [file 40462_2016_82_MOESM1_ESM.gif]

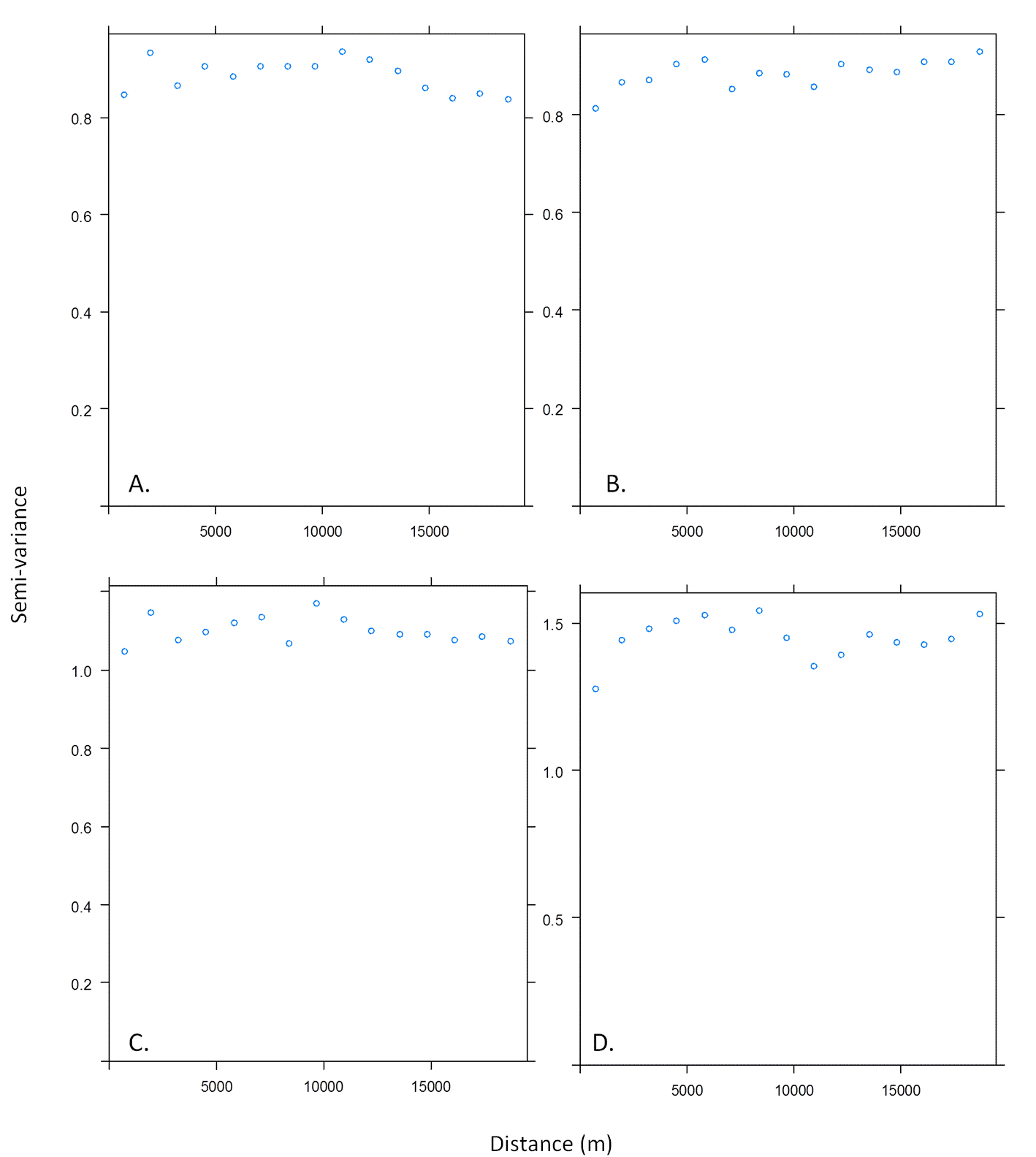

Supplement: Additional file 2: Figure S2. — Variograms showing variance of top model standardized residuals for A. average prey biomass (kg/km2) in the dry season, B. average biomass (kg/km2) in the wet season, C. frequency of prey occurrence in the dry season, and D. frequency of prey occurrence in the wet season. (GIF 40 kb) [file 40462_2016_82_MOESM2_ESM.gif]

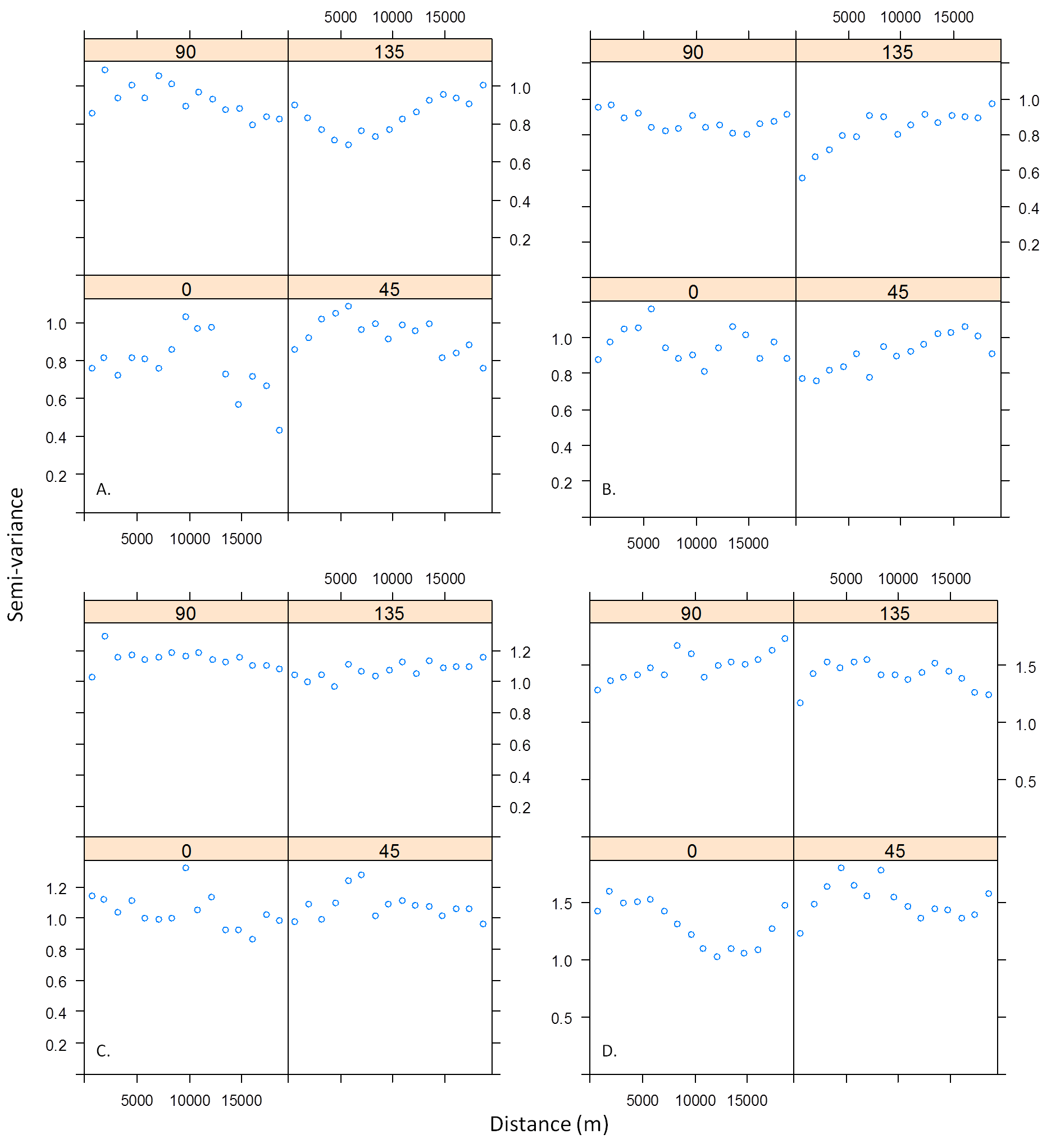

Supplement: Additional file 3: Figure S3. — Multi-directional variograms of standardized residuals for A. average prey biomass (kg/km2) in the dry season, B. average biomass (kg/km2) in the wet season, C. frequency of prey occurrence in the dry season, and D. frequency of prey occurrence in the wet season. Directions are indicated by degree values 0 = North-South, 45 = Northeast-Southwest, 90 = East-West and 135 = Southeast-Northwest. From the lack of strong spatial patters it appears that isotrophy is a reasonable assumption. (GIF 64 kb) [file 40462_2016_82_MOESM3_ESM.gif]

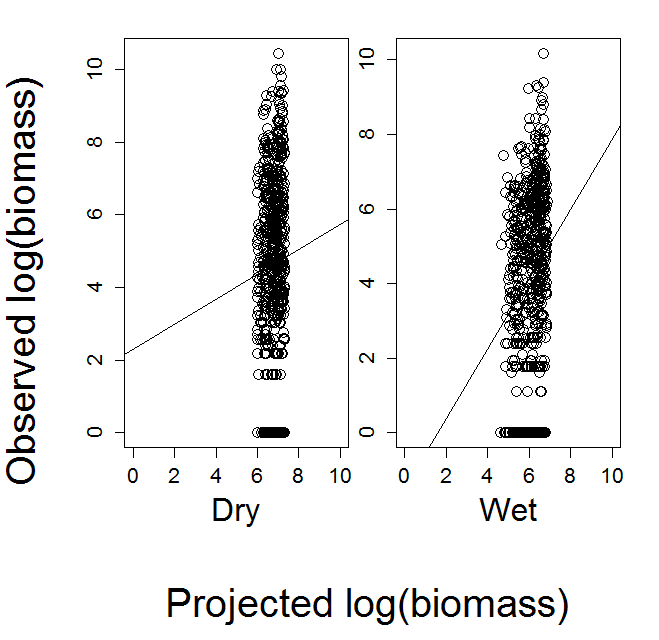

Supplement: Additional file 4: Figure S4. — Correlation between log of observed prey biomass (kg/km2) for each quadrat in the prey transects (N = 645) and (left) log of model-projected dry season prey biomass from the best dry season model (|r| = 0.11) and (right) log of model-projected wet season prey biomass (|r| = 0.15) from the best wet season model. (TIFF 1235 kb) [file 40462_2016_82_MOESM4_ESM.tiff]

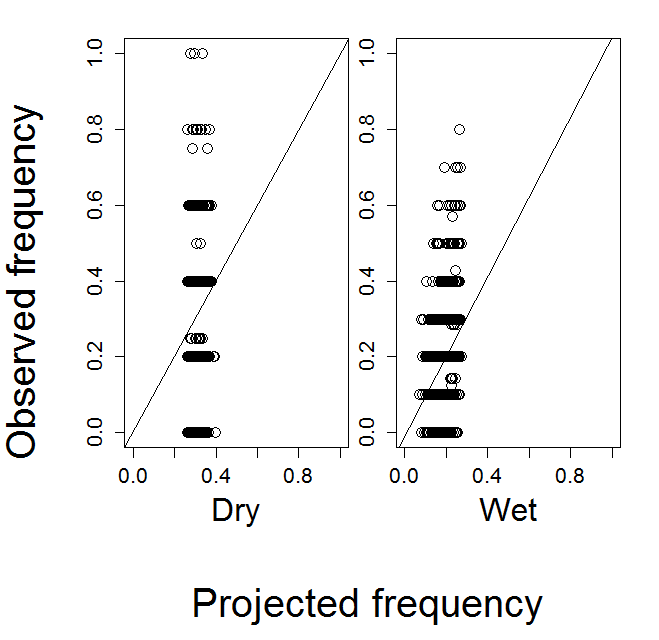

Supplement: Additional file 5: Figure S5. — Correlation between observed frequency (i.e. average probability that a quadrat is occupied by prey) for each quadrat in the prey transects (N = 645) and (left) log of model-projected dry season prey frequency from the best dry season model (|r| = 0.12) and (right) log of model-projected wet season prey frequency (|r| = 0.28) from the best wet season model. (TIFF 1235 kb) [file 40462_2016_82_MOESM5_ESM.tiff]

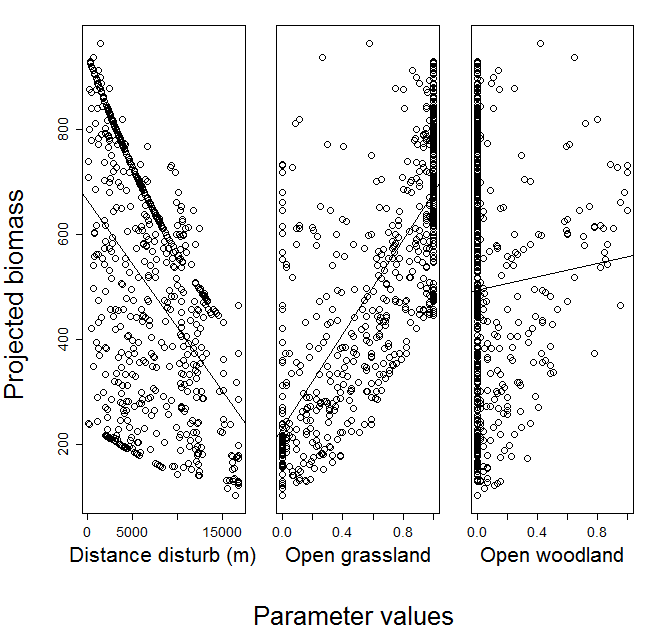

Supplement: Additional file 6: Figure S6. — Sensitivity analysis of input variables comprising the best wet season prey biomass model. The left graph shows prey biomass (kg/km2) values projected from the top model (Distance to disturbance + Open grassland + Open woodland) against the distance to disturbance input variable values (|r| = -0.46). The middle graph shows the same projected biomass values against the proportion of open grassland input variable values (|r| = 0.76). The right graph shows the same model output on the Y-axis against the proportion of open woodland input variable values (|r| = 0.06). (TIFF 1235 kb) [file 40462_2016_82_MOESM6_ESM.tiff]

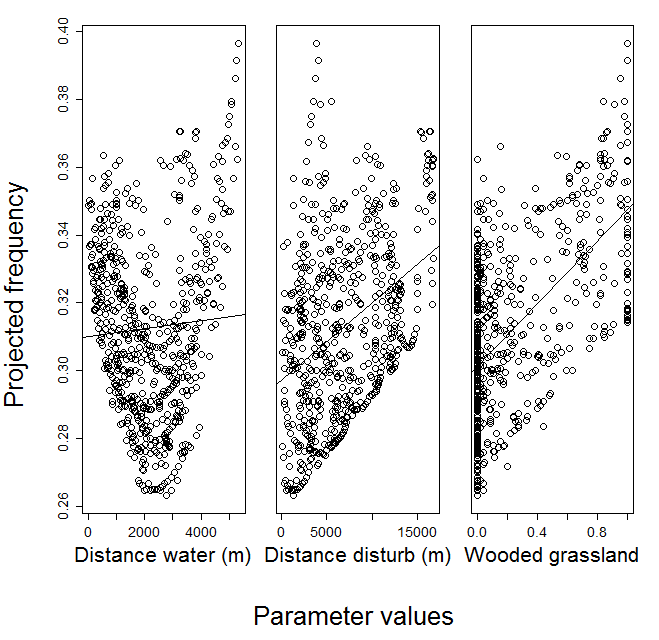

Supplement: Additional file 7: Figure S7. — Sensitivity analysis of input variables comprising the best dry season prey frequency model. The left graph shows prey frequency (average probability of occurrence) values projected from the top model (Distance to permanent water + Distance to permanent water2 + Distance to disturbance + Wooded grassland) against the distance to permanent water input variable values (|r| = 0.06). The middle graph shows the same projected frequency values against the distance to disturbance input variable values (|r| = 0.38). The right graph shows the same model output on the Y-axis against the proportion of wooded grassland input variable values (|r| = 0.59). (TIFF 1235 kb) [file 40462_2016_82_MOESM7_ESM.tiff]

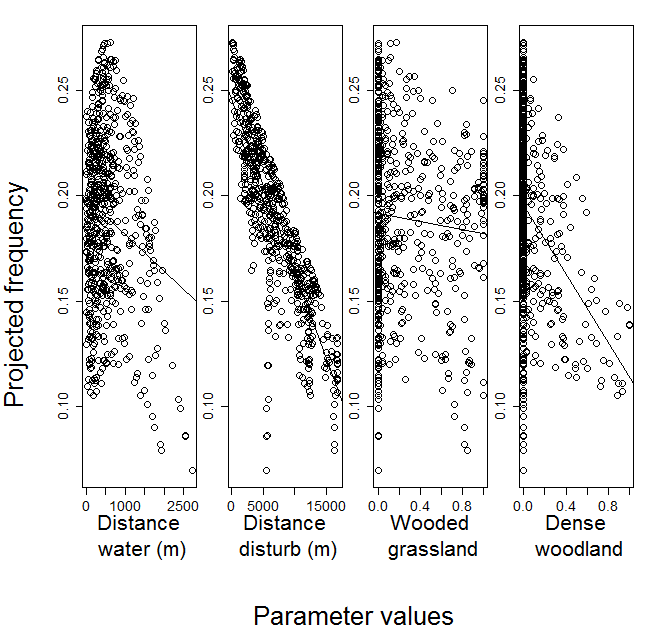

Supplement: Additional file 8: Figure S8. — Sensitivity analysis of input variables comprising the best wet season prey frequency model. The far left graph shows prey frequency (average probability of occurrence) values projected from the top model (Distance to all water + Distance to all water2 + Distance to disturbance + Wooded grassland + Dense woodland) against the distance to permanent water input variable values (|r| = -0.20). The inside left graph shows the same projected frequency values against the distance to disturbance input variable values (|r| = -0.87). The inside right graph shows the same model output on the Y-axis against the proportion of wooded grassland input variable values (|r| = -0.08). The far right graph shows the same projected frequency values against the proportion of dense woodland input variable values (|r| = -0.35). (TIFF 1235 kb) [file 40462_2016_82_MOESM8_ESM.tiff]

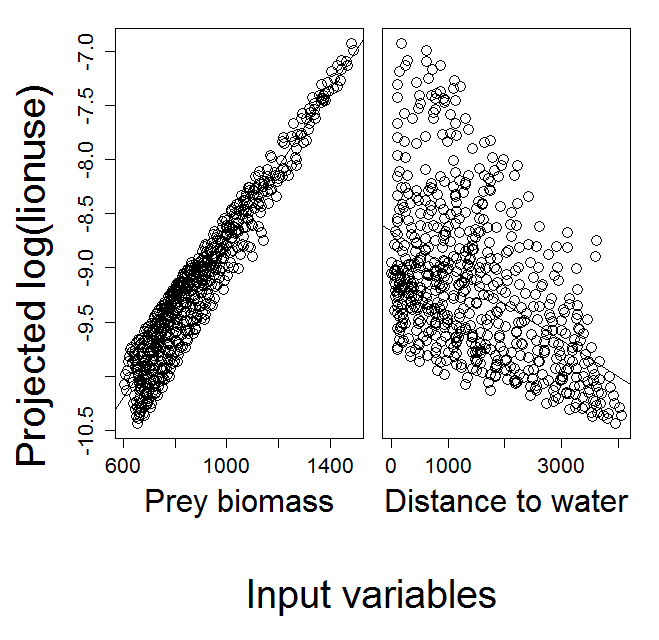

Supplement: Additional file 10: Figure S9. — Sensitivity analysis of input variables comprising the best dry season lion space use model. The left graph shows log(lionuse) values projected from the top model (Average prey biomass + Distance to permanent water) against the average prey biomass input variable values. The right graph shows the same model output on the Y-axis against the distance to permanent water input variable values. Both the visual pattern and the Pearson correlation coefficients (|r| = 0.89 and |r| = -0.33 respectively) indicate that average prey biomass is the more influential variable. (TIFF 1235 kb) [file 40462_2016_82_MOESM10_ESM.tiff]

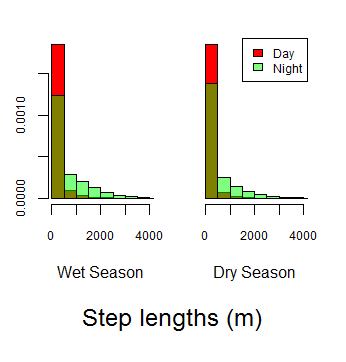

Supplement: Additional file 11: Figure S10. — Histograms showing 2 h step lengths (m) for all radio-collared lions (N = 6) in the study area between December 2009 and June 2011. The y-axis is a measure of relative frequency of occurrence so that seasons with different numbers of step lengths can be compared. In both the day (6:00 – 18:00) and night (18:00 – 6:00) lions moved longer distances more frequently in the wet season than the dry season with diurnal mean movement distance = 105 m during the dry season and 135 m during the wet season (t = -5.1; P < 0.0001) and nocturnal mean movement distance = 449 m during the dry season and 604 m during wet season (t = -10.5; P < 0.0001). During both diurnal and nocturnal periods 2-h movement distances were usually below 500 m. (TIFF 367 kb) [file 40462_2016_82_MOESM11_ESM.tiff]

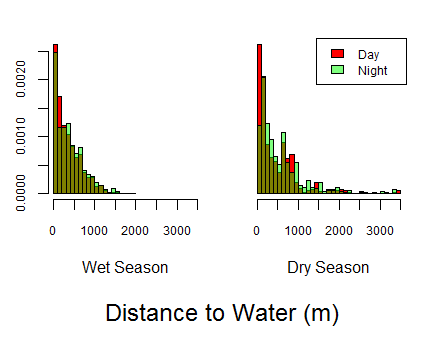

Supplement: Additional file 12: Figure S11. — Histograms showing average distance to water (m) for Kirawira pride between December 2009 and June 2011. The y-axis is a measure of the relative frequency of occurrence so that seasons with different numbers or re-locations can be compared. In both the wet and dry seasons lions preferred to be in close proximity to water sources. This preference was stronger in the daytime than nocturnally in both seasons, with the average dry season daytime distance = 463 m and average nighttime distance = 549 m, t = -3.314, P < 0.001) and average wet season daytime distance = 341 m and average nighttime distance = 386 m, t = -4.632, P < 0.0001). Pride lions were within 100 m of water more than 2x as frequently in the dry season daytime that dry season night. (PNG 4 kb) [file 40462_2016_82_MOESM12_ESM.png]

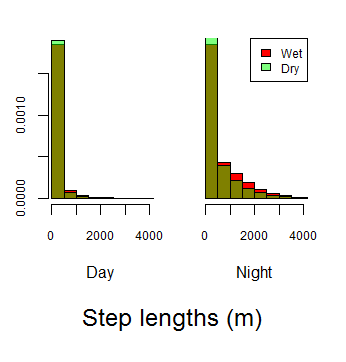

Supplement: Additional file 13: Figure S12. — Histograms showing 2 h step lengths (m) for all radio-collared lions (N = 6) in the study area between December 2009 and June 2011. The y-axis is a measure of relative frequency of occurrence so that seasons with different numbers of step lengths can be compared. In both the wet and dry seasons lions moved longer distances more frequently at night than during the day with wet season mean movement distance = 135 m during the day and 604 m during the night (t = -52.1; P < 0.0001) and dry season mean movement distance = 105 m during the day and 449 m during the night (t = -29; P < 0.0001). In both seasons 2-h movement distances were usually below 500 m. (TIFF 367 kb) [file 40462_2016_82_MOESM13_ESM.tiff]
